# Single-cell sequencing of the small and AT-skewed genome of malaria parasites

**DOI:** 10.1186/s13073-021-00889-9

**Published:** 2021-05-04

**Authors:** Shiwei Liu, Adam C. Huckaby, Audrey C. Brown, Christopher C. Moore, Ian Burbulis, Michael J. McConnell, Jennifer L. Güler

**Affiliations:** 1 Department of Biology, University of Virginia, Charlottesville, VA USA; 2 Division of Infectious Diseases and International Health, University of Virginia, Charlottesville, VA USA; 3 Department of Biochemistry and Molecular Genetics, University of Virginia School of Medicine, Charlottesville, VA USA; 4 Escuela de Medicina, Universidad San Sebastian, Puerto Montt, Chile; 5 Department of Neuroscience, University of Virginia School of Medicine, Charlottesville, VA USA; 6 Current address: Lieber Institute for Brain Development, Baltimore, MD USA

**Keywords:** Whole-genome amplification, AT-skewed genome, Malaria, Single-cell sequencing, MALBAC, Copy number variation, Single-nucleotide polymorphism

## Abstract

Single-cell genomics is a rapidly advancing field; however, most techniques are designed for mammalian cells. We present a single-cell sequencing pipeline for an intracellular parasite, *Plasmodium falciparum*, with a small genome of extreme base content. Through optimization of a quasi-linear amplification method, we target the parasite genome over contaminants and generate coverage levels allowing detection of minor genetic variants. This work, as well as efforts that build on these findings, will enable detection of parasite heterogeneity contributing to *P. falciparum* adaptation. Furthermore, this study provides a framework for optimizing single-cell amplification and variant analysis in challenging genomes.

**Supplementary Information:**

The online version contains supplementary material available at 10.1186/s13073-021-00889-9.

## Background

Malaria is a life-threatening disease caused by protozoan *Plasmodium* parasites. *P. falciparum* causes the greatest number of human malaria deaths [1]. The clinical symptoms of malaria occur when parasites invade human erythrocytes and undergo rounds of asexual reproduction by maturing from early-stage into late-stage forms and bursting from erythrocytes to begin the cycle again [2]. In this asexual cycle, parasites possess a single haploid genome during the early stages; rapid genome replication during subsequent stages leads to an average of 16 genome copies per late-stage individual [2].

Due to lack of effective vaccines, antimalarial drugs are required to treat malaria. However, drug efficacy is mitigated by the frequent emergence of resistant populations [3]. Both single-nucleotide polymorphisms (SNPs) and copy number variations (CNVs; the amplification or deletion of a genomic region) contribute to antimalarial resistance in *P. falciparum* [4–12]. It is important to assess genetic diversity within parasite populations to better understand the mechanisms of rapid adaption to antimalarial drugs and other selective forces. These studies are often complicated by multi-clonal infections and limited parasite material from clinical isolates.

Recent studies have begun to overcome these limitations for SNP analysis; methods including leukocyte depletion [13], selective whole-genome amplification (WGA) of parasite DNA [14], hybrid selection with RNA baits [15], and single-cell sequencing of *P. falciparum* parasites [16, 17] help enrich parasite DNA, determine genetic diversity, and understand the accumulation of SNPs in long-term culture. However, the study of genetic diversity in early stage parasites on a single-cell level remains challenging [16]; the lack of alternative single-cell approaches for *P. falciparum* parasites impedes the validation of SNP results by parallel investigations [18].

The dynamics of CNVs in evolving populations are not well understood. One reason for this is that the majority of *P. falciparum* CNVs have been identified by analyzing bulk DNA following selection, where CNVs are present in the majority of parasites [7, 9, 19–21]. However, many low frequency CNVs undoubtedly remain undetected. There is speculation that these low-frequency CNVs are either deleterious or offer no advantages for parasite growth or transmission [20, 22] but orthogonal methods to verify genome dynamics within the population are needed. Recent investigations in other organisms have analyzed single cells to detect low-frequency CNVs within heterogeneous populations [23–28].

Single-cell-based approaches provide a significant advantage for detecting rare genetic variants (SNPs and CNVs) by no longer deriving an average signal from large quantities of cells. However, short-read sequencing requires nanogram to microgram quantities of genomic material for library construction, which is many orders of magnitude greater than the genomic content of individual *Plasmodium* cells. Therefore, WGA is required to generate sufficient DNA quantities for these analyses. Several WGA approaches have been reported and each has advantages and disadvantages for different applications [29–32]; however, most have been optimized for mammalian cell analysis [30, 32–44]. Because WGA leads to high levels of variation in read abundance across the genome, CNV analysis in the single-cell context is especially challenging. Previous approaches have been tuned specifically for CNV detection in mammalian genomes, which are generally hundreds of kilobases to megabases in size [33, 39, 44–49].

To date, the detection of CNVs in single *P. falciparum* parasites using whole-genome sequencing has not been achieved. The application of existing WGA methods is complicated by the parasite’s small genome size and extremely imbalanced base composition (23 Mb haploid genome with 19.4% GC content [50]). Each haploid parasite genome contains 25 fg of DNA, which is ~ 280 times less than the ~ 6400 Mb diploid human genome. Therefore, an effective *P. falciparum* WGA method must be both highly sensitive and able to handle the extreme base composition. One WGA method, multiple displacement amplification (MDA), has been used to amplify single *P. falciparum* genomes with near complete genome coverage [16, 51]. These studies successfully detected SNPs in single parasite genomes but did not report CNV detection, which is possibly disrupted by low genome coverage uniformity [31] and the generation of chimeric reads by MDA [52], as well as the relatively small size of CNVs in *P. falciparum* (< 100 kb) [20, 22, 53, 54].

Multiple annealing and looping-based amplification cycles (MALBAC) is another WGA method that exhibits adequate uniformity of coverage, which was advantageous for detecting CNVs in single human cells [45]. MALBAC has the unique feature of quasi-linear pre-amplification, which reduces the bias associated with exponential amplification [45]. However, standard MALBAC is less tolerant to AT-biased genomes, unreliable with low DNA input, and prone to contamination [46, 55, 56]. Thus, optimization of this WGA method was necessary for *P. falciparum* genome analysis.

In this study, we developed a single-cell sequencing pipeline for *P. falciparum* parasites, which included efficient isolation of single parasite-infected erythrocytes, an optimized WGA step inspired by MALBAC, and a method of assessing sample quality prior to sequencing. We tested our pipeline on erythrocytes infected with laboratory-reared parasites as well as patient-isolated parasites with heavy human genome contamination. We assessed amplification bias first using a PCR-based approach and then by sequencing. We evaluated genome coverage breadth and coverage uniformity, as well as amplification reproducibility. Furthermore, we combined two approaches to limit false positives for CNV detection and applied stringent filtering steps for SNP detection in single-cell genomes. This work, as well as efforts that build on these findings, will enable the detection of parasite-to-parasite heterogeneity to clarify the role of genetic variations in the adaptation of *P. falciparum*. Furthermore, this study provides a framework for the optimization of single-cell whole genome amplification and CNV/SNP analysis in other organisms with challenging genomes.

## Methods

### Parasite culture

We freshly thawed erythrocytic stages of *P. falciparum* (*Dd2*, MRA-150, Malaria Research and Reference Reagent Resource Center, BEI Resources) from frozen stocks and maintained them as previously described [57]. Briefly, we grew parasites at 37 °C in vitro  at 3% hematocrit (serotype A positive human erythrocytes, Valley Biomedical, Winchester, VA) in RPMI 1640 medium (Invitrogen, USA) containing 24 mM NaHCO_3_ and 25 mM HEPES, and supplemented with 20% human type A positive heat inactivated plasma (Valley Biomedical, Winchester, VA) in sterile, plug-sealed flasks, flushed with 5% O_2_, 5% CO_2_, and 90% N_2_ [6]. We maintained the cultures with media changes every other day and sub-cultured them as necessary to keep parasitemia below 5%. We determined all parasitemia measurements using SYBR green-based flow cytometry [58]. We routinely tested cultures using the LookOut Mycoplasma PCR Detection Kit (Sigma-Aldrich, USA) to confirm negative infection status.

### Clinical sample collection

We obtained parasites from an infected patient admitted to the University of Virginia Medical Center with clinical malaria. The patient had a recent history of travel to Sierra Leone, a malaria-endemic country, and *P. falciparum* infection was clinically determined by a positive rapid diagnostic test and peripheral blood smear analysis. We obtained the sample of 1.4% early-stage parasites within 24 h of phlebotomy, incubated in the conditions described in *Parasite Culture* for 48 h, and washed the sample 3 times with RPMI 1640 HEPES to decrease levels of white blood cells. In order to fully evaluate our amplification method in the presence of heavy human genome contamination, we did not perform further leukodepletion. We set aside some of the sample for bulk DNA preparation (see “Bulk DNA extraction”). Using another portion of the sample, we enriched for parasite-infected erythrocytes using SLOPE (Streptolysin-O Percoll) method [59], which increased the parasitemia from 1.4 to 48.5% (Additional file 1: Figure S1). We then isolated the single *P. falciparum*-infected erythrocytes using the CellRaft AIR^TM^System (Cell Microsystems, Research Triangle Park, NC) as detailed in *Parasite Staining and Isolation*.

### Bulk DNA extraction

We lysed asynchronous *P. falciparum*-infected erythrocytes with 0.15% saponin (Sigma-Aldrich, USA) for 5 min and washed them with 1× PBS (diluted from 10× PBS Liquid Concentrate, Gibco, USA). We then lysed parasites with 0.1% Sarkosyl Solution (Bioworld, bioPLUS, USA) in the presence of 1 mg/ml proteinase K (from *Tritirachium album*, Sigma-Aldrich, USA) overnight at 37 °C. We extracted nucleic acids with phenol/chloroform/isoamyl alcohol (25:24:1) pH 8.0 (Sigma-Aldrich, USA) using 2 ml light Phase lock Gels (5Prime, USA). Lastly, we precipitated the DNA with ethanol using the standard Maniatis method [60].

### Parasite staining and isolation

For late-stage parasite samples, we obtained laboratory *Dd2* parasite culture with a starting parasitemia of 1.7% (60% early stage parasites). We separated late-stage *P. falciparum*-infected erythrocytes from non-paramagnetic early stages using a LS column containing MACS® microbeads (Miltenyi Biotec, USA, [61]). After elution of bound late-stage parasite, the sample exhibited a parasitemia of 80.8% (74.0% late-stage parasites, Additional file 1: Figure S1). For early-stage parasites, we obtained laboratory *Dd2* parasites culture with a starting parasitemia of 3% (46% early stage parasites). We harvested the non-paramagnetic early-stage parasites, which were present in the flow-through of the LS column containing MACS® microbeads. Next, we enriched the infected erythrocytes using the SLOPE method, which preferentially lysed uninfected erythrocytes [59]. The final parasitemia of enriched early-stage parasites was 22.8% (97.0% early-stage parasites, Additional file 1: Figure S1). To differentiate *P. falciparum*-infected erythrocytes from remaining uninfected erythrocytes or cell debris, we stained the stage-specific *P. falciparum*-infected erythrocytes with both SYBR green and MitoTracker Red CMXRos (Invitrogen, USA). We then isolated single *P. falciparum*-infected erythrocytes using the CellRaft AIR^TM^ System (Cell Microsystems, Research Triangle Park, NC). We coated a 100-micron single reservoir array (CytoSort Array and CellRaft AIR user manual, CELL Microsystems) with Cell-Tak Cell and Tissue Adhesive (Corning, USA) following the manufacturer’s recommendations. Then, we adhered erythrocytes onto the CytoSort array from a cell suspension of ~ 20,000 cells in 3.5 ml RPMI 1640 (Invitrogen, USA) with AlbuMAX II Lipid-Rich BSA (Thermo Fisher Scientific, USA) and Hypoxanthine (Sigma-Aldrich, USA). Lastly, we set up the AIR^TM^ System to automatically transfer the manually selected single infected erythrocytes (SYBR+, Mitotracker+) into individual PCR tubes.

### Steps to limit contamination

We suspended individual parasite-infected erythrocytes in freshly prepared lysis buffer, overlaid them with one drop (approx. 25 μl) of mineral oil (light mineral oil, BioReagent grade for molecular biology, Sigma Aldrich, USA), and stored them at − 80 °C until WGA. We amplified DNA in a clean positive pressure hood located in a dedicated room, using dedicated reagents and pipettes, and stored them in a dedicated box at − 20 °C. We wore a new disposable lab coat, gloves, and a face mask during reagent preparation, cell lysis, and WGA steps. We decontaminated all surfaces of the clean hood, pipettes, and tube racks with DNAZap (PCR DNA Degradation Solutions, Thermo Fisher Scientific, USA), followed by Cavicide (Metrex Research, Orange, CA), and an 80% ethanol rinse prior to each use. We autoclaved all tubes, tube racks, and the waste bin on a dry vacuum cycle for 45 min. Finally, we used sealed sterile filter tips, new nuclease-free water (Qiagen, USA) for each experiment, and filtered all salt solutions through a 30-mm syringe filter with 0.22 μm pore size (Argos Technologies, USA) before use in each experiment.

### Whole-genome amplification

#### Standard MALBAC

The MALBAC assay was originally designed for human cells [43, 45]. This approach involved making double-stranded DNA copies of genomic material using random primers that consist of 5 degenerate bases and 27 bases of common sequence. These linear cycles are followed by exponential amplification via suppression PCR. Here, we transferred individual cells into sterile thin-wall PCR tubes containing 2.5 μl of lysis buffer that yielded a final concentration of 25 mM Tris pH 8.8 (Sigma-Aldrich, USA), 10 mM NaCl (BAKER ANALYZED A.C.S. Reagent, J.T.Baker, USA), 10 mM KCl (ACS reagent, Sigma-Aldrich, USA), 1 mM EDTA (molecular biology grade, Promega, USA), 0.1% Triton X-100 (Acros Organics, USA), and 1 mg/ml Proteinase K (*Tritirachium album,* Sigma-Aldrich, USA). After overlaying one drop of mineral oil, we lysed cells at 50 °C for 3 h and inactivated the proteinase at 75 °C for 20 min, then 80 °C for 5 min before maintaining at 4 °C. We added 2.5 μl of amplification buffer to each sample to yield a final concentration of 25 mM Tris pH 8.8 (Sigma-Aldrich, USA), 10 mM (NH_4_)_2_SO_4_ (Molecular biology grade, Sigma-Aldrich, USA), 8 mM MgSO_4_ (Fisher BioReagents, Thermo Fisher Scientific, USA), 10 mM KCl (ACS reagent, Sigma-Aldrich, USA), 0.1% Triton X-100 (Acros Organics, USA), 2.5 mM dNTPs (PCR grade, Thermo Fisher Scientific, USA), 1 M betaine (PCR Reagent grade, Sigma-Aldrich, USA), and 0.667 μM of each random primer (5′GTGAGTGATGGTTGAGGTAGTGTGGAGNNNNNTTT 3′, and 5′GTGAGTGATGGTTGAGGTAGTGTGGAGNNNNNGGG 3′) ordered from Integrated DNA Technologies, USA. To denature DNA, we heated samples to 95 °C for 3 min and snap-cooled on an ice slush before gently adding 0.5 μl of enzyme solution (8000 U/ml *Bst* DNA Polymerase Large Fragment, New England Biolabs, USA, in 1× amplification buffer) into the aqueous droplet.

We thermo-cycled samples (Bio-Rad, USA) holding at 4 °C and heated according to the following cycles: 10 °C—45 s, 15 °C—45 s, 20 °C—45 s, 30 °C—45 s, 40 °C—45 s, 50 °C—45 s, 64 °C—10 min, 95 °C—20 s. The samples were immediately snap-cooled on an ice slush for at least 3 min to maintain the DNA in a denatured state for the next round of random priming. We added an additional 0.5 μl of enzyme solution and mixed thoroughly with a pipette on ice as above. We placed the samples back into the 4 °C thermo-cycler and heated according to the cycles listed above with an additional 58 °C step for 1 min before once again cooling on an ice slush for 3 min. We repeated the addition of enzyme mix (as above) and performed additional rounds of amplification cycles (as above, including the 58 °C step). Once completed, we placed the samples on ice and supplemented with cold PCR master mix to yield 50 μl with the following concentrations: 0.5 μM Common Primer (5′GTGAGTGATGGTTGAGGTAGTGTGGAG3′, Integrated DNA Technologies, USA), 1.0 mM dNTPs (PCR grade, Thermo Fisher Scientific, USA), 6.0 mM MgCl_2_ (Molecular biology, Sigma-Aldrich, USA), 1× Herculase II Polymerase buffer, and 1× Herculase II polymerase (Agilent Technologies, USA). We immediately thermo-cycled samples with the following temperature-time profile: 94 °C—40s, 94 °C—20s, 59 °C—20s, 68 °C—5 min, go to step two for several times, and an additional extended at 68 °C—5 min, and finally, a hold at 4 °C. For comparison, we used 18 or 19 linear cycles (for late- or early-stage parasites, respectively) and 17 exponential cycles for genomes amplified by the standard MALBAC protocol.

#### Optimized MALBAC

We made the following modifications to standard MALBAC to yield our optimized MALBAC protocol. (1) We froze cells at − 80 °C until usage because freeze-thaw enhanced cell lysis as previously reported [16]. (2) We removed betaine from the amplification buffer because it improved amplification of GC-rich sequences [62], which are infrequent in *P. falciparum* genomes (Additional file 2: Table S1). (3) We used a single random primer where the GC content of the degenerate bases were 20% instead of 50% (5′GTGAGTGATGGTTGAGGTAGTGTGGAGNNNNNTTT 3’) at final concentration of 1.2 μM. (4) We reduced the volume of the random priming reaction by added only 0.29 μl of 2× amplification buffer to the lysed samples and 0.13 μl of enzyme solution to the aqueous droplet each amplification cycle. (5) We added additional random priming cycles over prior MALBAC studies for a total of 18 (for late-stage parasites) or 19 (for early-stage parasites) cycles. (6) We reduced the total volume of exponential amplification from 50 to 20 μl and increased the number of exponential amplification cycles from 15 to 17. (7) We verified the presence of DNA products in the samples using the Qubit Fluorometer (1X dsDNA High-Sensitivity Assay Kit, Thermo Fisher Scientific, USA) before purifying nucleic acids by Zymo DNA Clean & Concentrator-5 (ZYMO Research).

### Pre-sequencing quality assessment

We assayed 6 distinct genomic loci across different chromosomes to determine variations in copy number following the whole-genome amplification step. We included this step, which employs highly sensitive droplet digital PCR (ddPCR, QX200 Droplet Digital PCRsystem, Bio-Rad, USA), to identify samples that exhibited more even genome coverage prior to short-read sequencing. The sequence of primers and probes are described in Additional file 2: Table S2 [6, 63, 64]. Each ddPCR reaction contained 5 μl of DNA (0.3 ng/μl for single-cell samples), 10 μl ddPCR Supermix for Probes (without dUTP), primers and probes with the final concentration in Additional file 2: Table S2, and sterile H_2_O to bring the per-reaction volume to 22 μl. We prepared droplets with the PCR mixture following the manufacturer’s protocol. After thermal cycling (95 °C—10 min; 40 cycles of 95 °C—30 s, 60 °C—60 s, and an infinite hold at 4 °C), we counted positive droplets using the Bio-Rad QX200 Droplet Reader (Bio-Rad, USA). We analyzed data using QuantaSoft (Bio-Rad, USA). For each gene, a no template control (sterile water, NTC) and a positive control (0.025 ng *Dd2* genomic DNA) are included in each ddPCR run. Following ddPCR, we calculated the “uniformity score” using the locus representation of the 6 genes: *seryl tRNA synthetase* (gene-1, PF3D7_0717700), *heat shock protein 70* (gene-2, PF3D7_0818900), *dihydrofolate reductase* (gene-3, PF3D7_0417200), *lactate dehydrogenase* (gene-4, PF3D7_1324900), *18S ribosomal RNA* (gene-5, PF3D7_0112300, PF3D7_1148600, PF3D7_1371000), and *multi-drug resistance transporter 1* (*Pfmdr1*, gene-6, PF3D7_0523000) in the amplified DNA sample relative to non-amplified DNA using the following equation:

$$ {\displaystyle \begin{array}{c}\mathrm{Uniformity}\ \mathrm{score}=\frac{\mathrm{gene}1}{\mathrm{gene}2}+\frac{\mathrm{gene}1}{\mathrm{gene}3}+\frac{\mathrm{gene}1}{\mathrm{gene}4}+\frac{\mathrm{gene}1}{\mathrm{gene}5}+\frac{\mathrm{gene}1}{\mathrm{gene}6}+\frac{\mathrm{gene}2}{\mathrm{gene}3}+\frac{\mathrm{gene}2}{\mathrm{gene}1}+\frac{\mathrm{gene}2}{\mathrm{gene}4}+\frac{\mathrm{gene}2}{\mathrm{gene}5}\\ {}+\frac{\mathrm{gene}2}{\mathrm{gene}6}+\frac{\mathrm{gene}3}{\mathrm{gene}4}+\frac{\mathrm{gene}3}{\mathrm{gene}1}+\frac{\mathrm{gene}3}{\mathrm{gene}2}+\frac{\mathrm{gene}3}{\mathrm{gene}5}+\frac{\mathrm{gene}3}{\mathrm{gene}6}+\frac{\mathrm{gene}4}{\mathrm{gene}5}+\frac{\mathrm{gene}4}{\mathrm{gene}1}+\frac{\mathrm{gene}4}{\mathrm{gene}2}+\frac{\mathrm{gene}4}{\mathrm{gene}3}+\frac{\mathrm{gene}4}{\mathrm{gene}6}\\ {}+\frac{\mathrm{gene}5}{\mathrm{gene}1}+\frac{\mathrm{gene}5}{\mathrm{gene}2}+\frac{\mathrm{gene}5}{\mathrm{gene}3}+\frac{\mathrm{gene}5}{\mathrm{gene}4}+\frac{\mathrm{gene}5}{\mathrm{gene}6}+\frac{\mathrm{gene}6}{\mathrm{gene}1}+\frac{\mathrm{gene}6}{\mathrm{gene}2}+\frac{\mathrm{gene}6}{\mathrm{gene}3}+\frac{\mathrm{gene}6}{\mathrm{gene}4}+\frac{\mathrm{gene}6}{\mathrm{gene}5}\end{array}} $$

When specific loci were over- or under-represented in the amplified sample, this score increased above the perfect representation of the genome; a uniformity score of 30 indicates that all genes are equally represented. We calculated the locus representation from the absolute copies of a gene measured by ddPCR from 1 ng of amplified DNA divided by the absolute copies from 1 ng of the bulk DNA control [65]. We only included samples in which all six genes were detected by ddPCR. The relative copy number of the *Pfmdr1*, which was amplified in the *Dd2* parasite line [66], was also used to track the accuracy of amplification. We calculated this value by dividing the ddPCR-derived absolute copies of *Pfmdr1* by the average absolute copies of the 6 assayed loci (including *Pfmdr1*, normalized to a single copy gene*)*. To confirm the efficiency of ddPCR detection as a pre-sequencing quality control step, we determined the strength of association based on the pattern of concordance and discordance between the ddPCR detection and the sequencing depth of the 5 gene targets with Kendall rank correlation (*18S ribosomal RNA* was excluded from correlation analysis due to the mapping of non-unique reads). We then calculated the correlation coefficient (Additional file 2: Table S3). When the level of ddPCR detection corresponded to the sequencing depth in at least 3 of the 5 gene targets (a correlation coefficient of > 0.6), we regarded the two measurements as correlated.

### Short-read sequencing

We fragmented MALBAC amplified DNA (> 1 ng/μL, 50 μL) using Covaris M220 Focused Ultrasonicator in microTUBE-50 AFA Fiber Screw-Cap tubes (Covaris, USA) to a target size of 350 bp using a treatment time of 150 s. We determined the fragment size range of all sheared DNA samples (291–476 bp) with a Bioanalyzer using High Sensitivity DNA chips (Agilent Technologies, USA). We used the NEBNext Ultra DNA Library Preparation Kit (New England Biolabs, USA) to generate Illumina sequencing libraries from sheared DNA samples. Following adaptor ligation, we applied 3 cycles of PCR enrichment to ensure that sequences contained both adapters and ranged in size from 480 to 655 bp. We quantified the proportion of adaptor-ligated DNA using real-time PCR and combined equimolar quantities of each library for sequencing on 4 lanes of a Nextseq 550 (Illumina, USA) using 150 bp paired-end cycles. We prepared the sequencing library of clinical bulk DNA as above except that it was sequenced it on a Miseq (Illumina, USA) using 150 bp paired-end sequencing.

### Sequencing analysis

We performed read quality control steps and sequence alignments essentially as previously described [53] (Additional file 1: Figure S2A). Briefly, we removed Illumina adapters and PhiX reads and trimmed MALBAC common primers from reads with BBDuk tool in BBMap [67]. To identify the source of DNA reads, we randomly subsetted 10,000 reads from each sample by using the reformat tool in BBMap [67] and blasted reads in nucleotide database using BLAST+ remote service. We aligned each fastq file to the hg19 human reference genome and kept the unmapped reads (presumably from *P. falciparum*) for analysis. Then, we aligned each fastq file to the *3D7 P. falciparum* reference genome with Speedseq [68]. We discarded the reads with low-mapping quality score (below 10) and duplicated reads using Samtools [69]. To compare the coverage breadth (the percentage of the genome that has been sequenced at a minimum depth of one mapped read, [70]) between single-cell samples, we extracted mappable reads from BAM files using Samtools [69] and randomly downsampled to 300,000 reads using the reformat tool in BBMap [67]. This read level was dictated by the sample with the lowest number of mappable reads (ENM, Additional file 2: Table S4). We calculated the coverage statistics using Qualimap 2.0 [71] for the genic, intergenic, and whole genome regions.

To understand where the primers of MALBAC amplification are annealing to the genome, we overlaid information on the boundaries of genic or intergenic regions with the mapping position of reads containing the MALBAC primer common sequence. To do so, we kept the MALBAC common primers in the sequencing reads, filtered reads, and aligned reads as in the above analysis. We subsetted BAM files for genic and intergenic regions using Bedtools, searched for the MALBAC common primer sequence using Samtools, and counted reads with MALBAC common primer using the pileup tool in BBMap (Additional file 2: Table S5).

We conducted single-cell sequencing analysis following the steps in Additional file 1: Figure S2B. We compared the variation of normalized read abundance (log_10_ ratio) at different bin sizes using boxplot analysis (R version 3.6.1) and determined the bin size of 20 kb using the plateau of decreasing variation of normalized read abundance (log_10_ ratio) when increasing bin sizes. We then divided the *P. falciparum* genome into non-overlapping 20 kb bins using Bedtools [72]. The normalized read abundance was the mapped reads of each bin divided by the total average reads in each sample. To show the distribution of normalized read abundance along the genome, we constructed circular coverage plots using Circos software and ClicO FS [73, 74]. To assess uniformity of amplification, we calculated the coefficient of variation of normalized read abundance by dividing the standard deviation by the mean and multiplying by 100 [31, 75] and analyzed the equality of coefficients of variation using the R package “cvequality” version 0.2.0 [76]. We employed correlation coefficients to assess amplification reproducibility as previous studies [77]. Due to presence of non-linear correlations between some of the samples, we used Spearman correlation for this analysis. We removed outlier bins if their read abundance was above the highest point of the upper whisker (Q3 + 1.5 × interquartile range*)* or below the lowest point of the lower whisker (Q1-1.5 × interquartile range) in each sample. Then, we subsetted remaining bins present in all samples to calculate the correlation coefficient using the R package “Hmisc” version 4.3-0 [78]. We visualized Spearman correlations, histograms, and pairwise scatterplots of normalized read abundance using “pairs.panels” in the “psych” R package. We then constructed heatmaps and hierarchical clustering of Spearman correlation coefficient with the “gplots” R package version 3.0.1.1 [79]. Additionally, to estimate the chance of random primer annealing during MALBAC pre-amplification cycles (likely affected by the GC content of genome sequence), we generated all possible 5-base sliding windows with 1 base step-size in the *P. falciparum* genome and calculated the GC content of the 5-base windows using Bedtools (Additional file 2: Table S1) [72].

### Copy number variation analysis

We conducted CNV analysis following the steps in Additional file 1: Figure S2C. To ensure reliable CNV detection, our CNV analysis is limited to the core genome, as defined previously [80]. Specifically, we excluded the telomeric, sub-telomeric regions, and hypervariable *var* gene clusters, due to limited mappability of these regions. For discordant/split read analysis, we used LUMPY [81] in Speedseq to detect CNVs (> 500 bp) with at least two supporting reads in each sample (Additional file 2: Table S6). For read-depth analysis, we further filtered the mapped reads using a mapping quality score of 30. We counted the reads in 1 kb, 5 kb, 8 kb, and 10 kb bins by Bedtools and we used Ginkgo [82] to normalize (by dividing the count in each bin by the mean read count across all bins), correct the bin read counts for GC bias, independently segment (using a minimum of 5 bins for each segment), and determine the copy number state in each sample with a predefined ploidy of 1 ([82], Additional file 2: Table S7). The quality control steps of Ginkgo were replaced by the coefficient of variation of normalized read count used in this study to assess uniformity in each cell. Lastly, we identified shared CNVs from the two methods when one CNV overlapped with at least 50% of the other CNV and vice versa (50% reciprocal overlap). We calculated precision of CNV detection in single-cell genome by dividing the number of true CNVs (same as those detected in the bulk sample) by the total number of CNVs. We calculated sensitivity by dividing the number of true CNVs by 3 (total number of true CNVs in the bulk sample).

### Single-nucleotide polymorphism analysis

We conducted SNP analysis following the MalariaGen *P. falciparum* Community Project V6.0 pipeline [83, 84] based on GATK best practices [85–87]. We first applied GATK’s Base Quality Score Recalibration using default parameters. We used GATK’s HaplotypeCaller to detect potential SNPs in BAM files and genotyped them using GATK’s CombineGVCFs and GenotypeGCVFs. We ran GATK’s VariantRecalibrator using previously validated SNP set from the *Pf*-Crosses variant set as a training set [88]. We then applied GATK’s ApplyRecalibration to assign each SNP a variant quality score log-odds (VQSLOD) quality score, which uses a machine learning approach to assess the probability that raw SNPs are true variants based on the training set. Higher VQSLOD scores indicate higher quality SNPs; filtering SNPs by “VQSLOD score > 0” has been applied to variant detection studies using the GATK pipeline [51, 83, 89], whereas VQSLOD score > 6 is recommended to further improve SNP accuracy in *P. falciparum* specifically [83]. We calculated precision by dividing the number of called SNP variants with the same genotype as the standard data set (SNPs detected in the *Dd2* bulk sample) by the total number of SNP variants called in each single-cell sample. We calculated sensitivity by dividing the number of called SNP variants with the same genotype as the standard SNPs in single-cell samples by the number in the bulk standard SNPs at three different stringency levels: VQSLOD score > 0, VQSLOD score > 6, and VQSLOD score > 6 with read depth > 10. We only included bi-allelic SNPs (loci with either the wild type or one mutant type allele) from the core genome in this analysis [83]. We also evaluated the detection of SNPs in resistant genes of the *Dd2* parasite line. We successfully detected 16 out of 17 resistant SNPs in the bulk sample at VQSLOD > 6; the one remaining SNP failed to pass the filtering step (VQSLOD = 3.77) so we excluded it from all single-cell analyses. We further filtered novel SNPs in single-cell samples by removing those that exhibited multiple alleles (mixed allele SNPs). We utilized SnpEff [90] to annotate VCF files and used VIVA (v0.4.0) [91] to generate heatmaps to illustrate the relationship between SNP calling and read depth.

## Results

### *Plasmodium falciparum* genomes from single-infected erythrocytes are amplified by MALBAC

**Fig. 1:**
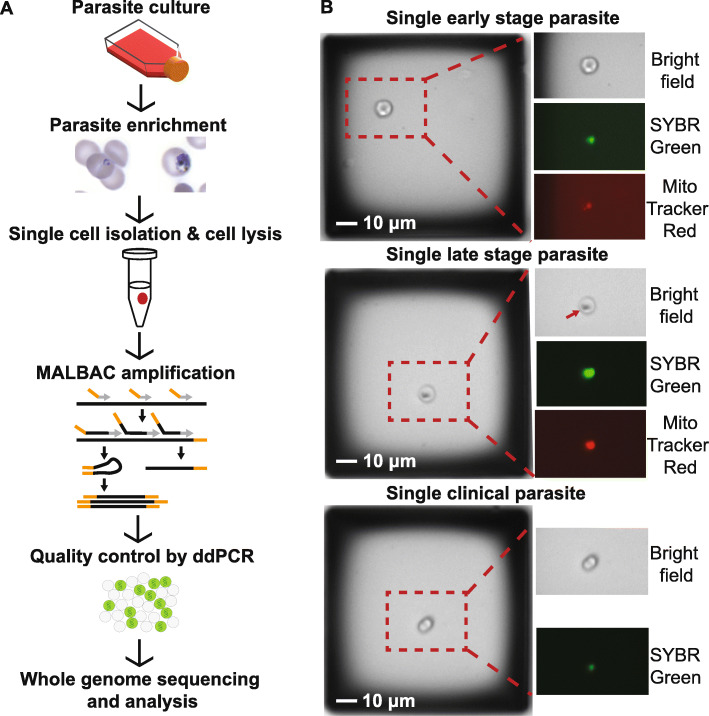
Single *P. falciparum*-infected erythrocytes are isolated, amplified, and sequenced. **a** Experimental workflow. Parasites are grown in vitro in human erythrocytes or isolated from infected patients. To limit the number of uninfected erythrocytes in the sample, infected cells are enriched using column and gradient-based methods (see “Methods”). Individual early-stage (left image) and late-stage (right image) parasite-infected erythrocytes were automatically isolated into PCR tubes using the CellRaft AIR System (Cell Microsystems, see panel **b**). All cells were lysed and amplified by MALBAC. MALBAC uses a combination of common (orange) and degenerate (grey) primers to amplify the genome. The quality of amplified genomes was assessed prior to library preparation and sequencing using droplet digital PCR (ddPCR); DNA is partitioned into individual droplets to measure gene copies. Samples were Illumina sequenced and analyzed as detailed in Additional file 1: Figure S2. **b** Parasite stage visualization on the CellRaft AIR System using microscopy (× 10 magnification). Enriched early- and late-stage parasite*-*infected erythrocytes at low density were seeded into microwells to yield only a single cell per well (left image of each group), and identified with SYBR green and Mitotracker Red staining (indicates parasite DNA and mitochondrion, respectively). Early-stage parasites exhibited lower fluorescence due to their smaller size, and late-stage parasites had noticeable dark spots (arrow) due to the accumulation of hemozoin pigment. Scale bar represents 10 μm.

Our single-cell sequencing pipeline for *P. falciparum* parasites included stage-specific parasite enrichment, isolation of single infected erythrocytes, cell lysis, whole genome amplification, pre-sequencing quality control, whole genome sequencing, and analysis steps (Fig. 1a). We collected parasites from either an in vitro-propagated laboratory line (*Dd2*) or from a blood sample of an infected patient (referred to as “laboratory” and “clinical” parasites, respectively). This allowed us to test the efficiency of our procedures on parasites from different environments with varying amounts of human host DNA contamination. Furthermore, for laboratory *Dd2* parasite samples, we isolated both early- (1n) and late- (~16n) stage parasite-infected erythrocytes to evaluate the impact of parasite DNA content on the performance of WGA. For single-cell isolation, we used the microscopy-based CellRaft Air System (Fig. 1b), which has the benefit of low capture volume (minimum: 2 μl) and visual confirmation of parasite stages. Following isolation, using the standard MALBAC protocol (termed *n*on-optimized *M*ALBAC), we successfully amplified 3 *e*arly-stage (ENM) and 4 *l*ate-stage (LNM) laboratory *Dd2* parasite samples. We also applied a version of MALBAC that we optimized for the small AT-rich *P. falciparum* genome (termed *o*ptimized *M*ALBAC) to 42 *e*arly- (EOM) and 20 *l*ate-stage (LOM) laboratory *Dd2* parasite samples as well as 4 *c*linical samples (COM) (Additional file 2: Table S8). Compared to standard MALBAC, our optimized protocol has a lower reaction volume, more amplification cycles, and a modified pre-amplification random primer (see “Methods” for more details). Using this method, we successfully amplified 43% of the early- and 90% of the late-stage laboratory *Dd2* parasite samples and 100% of the clinical samples (see post-amplification yields in Additional file 2: Tables S8 and S9).

### Pre-sequencing quality control step identifies samples with more even genome amplification

We assessed the quality of WGA products from single cells using droplet digital PCR (ddPCR) to measure the copy number of known single- and multi-copy genes dispersed across the *P. falciparum* genome (6 genes in total including *Pfmdr1*, which is present at ~ 3 copies in the *Dd2* laboratory parasite line). Using this sensitive quantitative method, along with calculation of a “uniformity score”, which reflects both locus dropout and over-amplification, we were able to select genomes that had been more evenly amplified; a low uniformity score and accurate copy number value indicated a genome that had been amplified with less bias (see “Methods” for details on score calculation and Additional file 2: Table S10 for raw data). This quality control step was important to reduce unnecessary sequencing costs during single-cell studies.

When we analyzed differences between amplified samples by optimized MALBAC (17 EOM samples and 14 LOM samples processed for ddPCR evaluation) and non-optimized MALBAC (3 ENM and 4 LNM samples), we found that samples amplified with the optimized protocol were generally more evenly covered than those from the standard method (Table 1). Specifically, one ENM sample lacked detection of any of the target genes (likely due to heavy contamination from non-parasite DNA) and other ENM and LNM samples consistently showed over-amplification of a set of 2 genes (*P. falciparum* seryl-tRNAsynthetase and 18S ribosomal RNA; Additional file 2: Table S10). Therefore, due to evidence of a high level of bias in the majority of ENM/LNM samples, we selected the ENM and LNM samples (one each) with the lowest level of ddPCR-based bias for sequencing. We also used ddPCR results to select 13 EOM and 10 LOM samples for sequencing (Additional file 2: Table S8). Overall, selected samples had lower average uniformity scores (i.e., 248 and 1012 for selected and unselected EOMs, respectively, see Table 1). For clinical parasite samples, 3 out of 4 COM samples showed a lack of ddPCR detection on at least one parasite gene; thus, we were not able to calculate uniformity scores for these samples and the amplification of clinical genomes was likely more skewed than laboratory samples (Table 1).

**Table 1:** Pre-sequencing quality control by droplet digital PCR

Result	Sample type	MALBAC type	Sample name (#)	Pre-sequencing ddPCR assessment	
				Uniformity score AVG (SD)*	*Pfmdr1* CN AVG (SD)
Sequenced	Single cell	Optimized	EOM (13)	248 (202)	2.6 (0.8)
			LOM (10)	118 (69)	2.2 (1.3)
			COM (2)	369 (–)	1.9 (0.8)
		Non-optimized	ENM (1)	18519 (–)	0.2 (–)
			LNM (1)	13121 (–)	0.1 (–)
	Bulk	N/A	*Dd2*_bulk (1)	30	2.7
			Clinical_bulk (1)	–	–
Not sequenced	Single cell	Optimized	EOM (4)	1012 (195)	3.7 (3.9)
			LOM (4)	775 (683)	2.8 (2.1)
			COM (2)	–^ (–)	4.7 (6.6)
		Non- optimized	ENM (2)	13689 (–)	0 (–)
			LNM (3)	1578 (–)	0.1 (0.1)

*EOM* early-stage single parasites amplified by optimized MALBAC, *LOM* late-stage single parasites amplified by optimized MALBAC, *COM* clinical single parasites amplified by optimized MALBAC, *ENM* early-stage single parasites amplified by non-optimized MALBAC, *LNM* late-stage single parasites amplified by non-optimized MALBAC, *AVG* average, *SD* standard deviation

*Uniformity scores were calculated when all of the six genes were detected by ddPCR in the sample

^Due to the lack of ddPCR detection of some genes in COM samples, the uniformity score could not be calculated. (–) Indicates only one sample was included in the calculation

Both standard and optimized MALBAC-amplified parasite genomes were short-read sequenced alongside a matched bulk DNA control (Table 1). To confirm the efficiency of ddPCR detection as a pre-sequencing quality control step, we calculated the correlation between ddPCR quantification and the sequencing depth at these specific loci in each sample. We found that the ddPCR-derived gene copy concentration was correlated with sequencing coverage of the corresponding genes in many samples (Additional file 2: Table S3, 17 out of 28 samples with a Kendal rank correlation coefficient ≥ 0.6), confirming the validity of using ddPCR detection as a quality control step prior to sequencing.

### Optimized MALBAC limits contamination of single-cell genomes

**Fig. 2:**
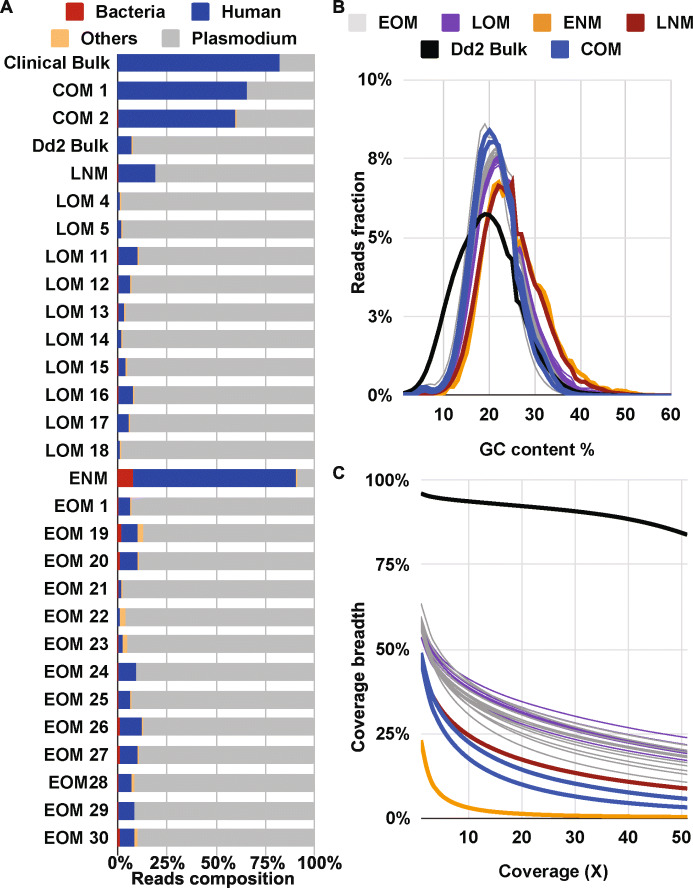
Sequencing statistics show benefits of optimized MALBAC. **a** Contribution of reads based on organism type.  A subset of 10,000 reads from each sample were randomly selected for BLAST to identify sources of DNA. Color representation: bacteria (red); human (blue); other organisms (orange); *Plasmodium* (grey). **b** GC content of *P. falciparum* mapped reads. GC content of reads was calculated by Qualimap. Color representation: EOM (grey): early-stage single parasites amplified by optimized MALBAC; LOM (purple): late-stage single parasites amplified by optimized MALBAC; ENM (orange): early-stage single parasites amplified by non-optimized MALBAC; LNM (dark red): late-stage single parasites amplified by non-optimized MALBAC; *Dd2* bulk genomic DNA (black); COM samples (blue): clinical single parasites amplified by optimized MALBAC. Clinical bulk genomic DNA is not shown here due to < 1% of the genome being covered by at least one read. **c** Fraction of *P. falciparum* genome covered by at least 1 read. The fraction of the genome was calculated by Qualimap. Color representations are the same as described in panel **b**

After read quality control steps, we mapped the reads to the *P. falciparum 3D7* reference genome (see “Methods” and Additional file 1: Figure S2 for details). We first assessed the proportion of contaminating reads in our samples; NCBI Blast results showed that the majority of non-*P. falciparum* reads were of human origin (Fig. 2a). The mean proportions of human reads in EOM samples (6.6%, SD of 3.2%) and LOM samples (4.3%, SD of 2.9%) were similar to that in the control bulk sample (7.4%, Fig. 2a); in fact, a majority of optimized MALBAC samples were lower than the bulk level (14/23). Conversely, the proportion of human reads in ENM and LNM samples were substantially higher (81.8% and 18.9%, respectively). As shown in other studies [92, 93], the clinical bulk DNA (81.9%) contained a much higher level of human contamination than the laboratory *Dd2* bulk DNA (7.4%). However, we found that the proportion of the human contaminating DNA in the two single-cell COM samples was considerably lower than the comparable bulk value (58.8% and 65.5%). The second most common source of contaminating reads was from bacteria such as *Staphylococcus* and *Cutibacterium*. The ENM sample exhibited a ~ 10-fold increase in the proportion of bacterial reads over averaged EOM samples (8.2% versus 0.8%, respectively) whereas the LNM samples showed the same proportion of bacterial reads as the averaged LOM samples (0.2%). These results indicated that the optimized MALBAC protocol exhibits lower amplification bias towards contaminating human and bacterial DNA in *P. falciparum* samples.

### Amplification bias and uniformity is altered in single-cell genomes

To further assess the optimized MALBAC protocol, we evaluated GC bias at several steps of our pipeline (i.e., WGA, library preparation, and the sequencing platform). Analysis of the bulk genome control (without WGA) indicated that there was little GC bias introduced by the library preparation, sequencing, or genome alignment steps; the GC content of mapped reads from bulk sequencing data is 18.9% (Table 2), which was in line with the GC content (19.4%) of the reference genome [50]. We then compared values from single-cell samples to those from the appropriate bulk control to evaluate the GC bias caused by MALBAC amplification (Fig. 2b). The average GC content of all EOM (21.4%), LOM (22.4%), and COM (20.7%) samples was within 1–3.5% of the bulk controls from laboratory *Dd2* and clinical samples (18.9% and 19.7%, respectively, Table 2). However, the average GC content of ENM and LNM samples was 6.1% and 5.4% greater than that of the bulk control; this result is consistent with the high GC preference of the standard MALBAC protocol [30, 46]. ENM and LNM samples also showed a greater proportion of mapped reads with high GC content (> 30%) than EOM, LOM, and bulk DNA samples (Fig. 2b).

**Table 2:** Average GC-content and coverage breadth of sequenced samples

Reads	Sample name (#)	Average of mean coverage (X)	Average GC content	Average coverage breadth		
				Whole genome	Genic regions	Intergenic regions
All mappable reads	EOM (13)	37.54	21.4%	57.9%	78.0%	27.8%
	LOM (10)	43.10	22.4%	57.3%	79.0%	25.0%
	COM (2)	9.54	20.7%	48.0%	67.7%	18.5%
	ENM (1)	1.47	25.0%	23.0%	34.4%	6.1%
	LNM (1)	20.43	24.3%	47.4%	67.9%	16.9%
	*Dd2*_bulk (1)	75.83	18.9%	96.1%	97.0%	94.9%
	Clinical_bulk (1)	0.03	19.7%	0.3%	0.3%	0.2%
Down-sampled*	EOM (13)	1.66	21.4%	30.9%	47.2%	6.7%
	LOM (10)	1.69	22.4%	32.1%	49.8%	5.8%
	COM (2)	1.66	20.8%	31.1%	47.0%	7.5%
	ENM (1)	1.33	25.2%	21.7%	32.9%	5.0%
	LNM (1)	1.62	24.3%	26.2%	40.3%	5.1%
	*Dd2*_bulk (1)	1.85	18.8%	76.8%	80.6%	71.2%

*Down-sampling is to 300,000 mappable reads based on the sample with the lowest number of mappable reads (ENM)

Since GC bias during the amplification step can limit which areas of the genome are sequenced, we assessed the genome coverage of MALBAC-amplified samples. The coverage breadth of single-cell samples increased by 34.9% in early-stage samples (Fig. 2c, orange-ENM to grey-EOM lines) and by 9.9% for late-stage samples following optimization (Fig. 2c, red-LNM to purple-LOM lines, see values in Table 2). Despite just a single ENM and LNM sample for comparison, the variation of coverage breadth across all EOM/LOM samples is low (Table S4, SD of 1.9%), indicating that differences between the two methods are substantial. This pattern of differences is conserved despite random down-sampling of reads to the same number per sample (300,000; Table 2).

Even though optimized MALBAC showed less bias towards GC-rich sequences, it was still problematic for highly AT-rich and repetitive intergenic regions (mean of 13.6% GC content, [50]). The fraction of intergenic regions covered by reads was only 27.8% for EOM samples and 25.0% for LOM samples on average. When we excluded intergenic regions, the fraction of genic regions of the genome covered by at least one read reached an average of 78.0% and 79.0% for EOM and LOM samples (Table 2). Conversely, the coverage of both intergenic and genic regions was substantially lower for the non-optimized samples. Coverage of the *P. falciparum* genome in the clinical bulk sample was very low due to heavy contamination with human reads (0.3% of the genome was covered by at least one read). This was much lower than that from the 2 COM samples (an average of 48%, Fig. 2c and Table 2).

**Fig. 3:**
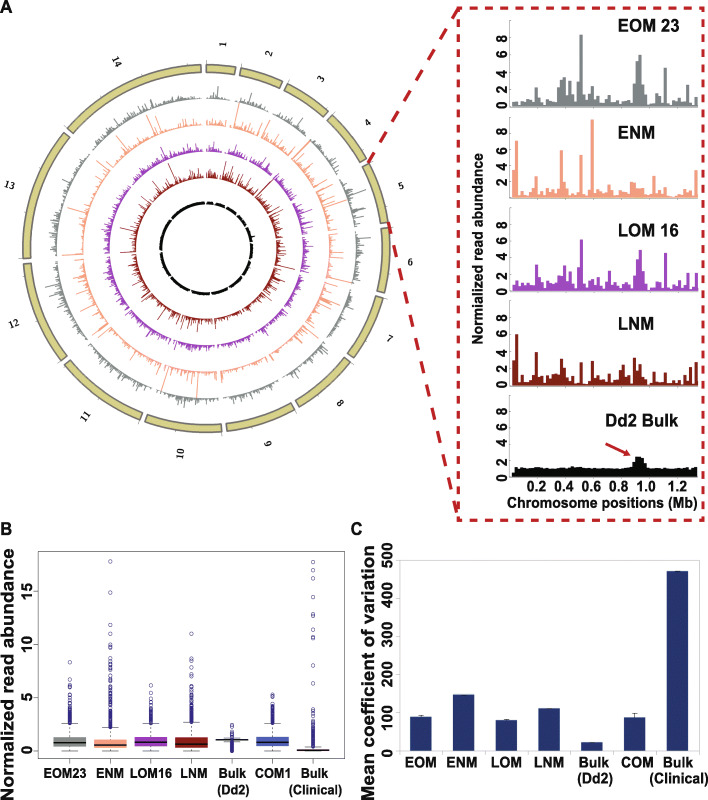
Samples amplified by optimized MALBAC display improved uniformity of read abundance. **a** Normalized read abundance across the genome. The reference genome was divided into 20-kb bins and read counts in each bin were normalized by the mean read count in each sample. The circles of the plot represent (from outside to inside): chromosomes 1 to 14 (tan); one EOM sample (#23, grey); one ENM sample (#3, orange); one LOM sample (#16, purple); one LNM sample (#2, dark red); *Dd2* bulk genomic DNA (black). The zoomed panel shows the read distribution across chromosome 5, which contains a known CNV (*Pfmdr1* amplification, arrow on *Dd2* bulk sample). **b** Distribution of normalized read abundance values for all bins. The boxes were drawn from Q1 (25th percentiles) to Q3 (75th percentiles) with a horizontal line drawn in the middle to denote the median of normalized read abundance for each sample. Outliers, above the highest point of the upper whisker (Q3+ 1.5 × IQR) or below the lowest point of the lower whisker (Q1−1.5 × IQR), are depicted with circles. One sample from each type is represented (see all samples in Additional file 1: Figure S3C). **c** Coefficient of variation of normalized read abundance. The average and SD (error bars) coefficient of variation for all samples from each type is represented (EOM: 13 samples; ENM: 1 sample; LOM: 10 samples; LNM: 1 sample; *Dd2* bulk: 1 sample; COM: 2 samples; Clinical bulk: 1 sample). See “Methods” for calculation

To investigate the uniformity of read abundance distributed over the *P. falciparum* genome, we divided the reference genome into 20-kb bins and plotted the read abundance in these bins over the 14 chromosomes (Fig. 3a, Additional file 1: Figures S3 and S4A). We selected a 20-kb bin size based on its relatively low coverage variation compared to smaller bin sizes and similar coverage variation as the larger bin sizes (Additional file 1: Figure S5). To quantitatively measure this variation, we normalized the read abundance per bin in each sample by dividing the raw read counts with the mean read counts per 20-kb bin (Fig. 3b, Additional file 1: Figure S3C). Here, the bulk control displayed the smallest range of read abundance for outlier bins (blue circles) and lowest interquartile range (IQR) value of non-outlier bins (black box, Fig. 3b, Additional file 1: Figure S3C), indicating less bin-to-bin variation in read abundance. Both EOM and LOM samples exhibited a smaller range of normalized read abundance in outlier bins than ENM and LNM samples (Fig. 3b, Additional file 1: Figure S3C). In addition, the read abundance variation of COM samples was similar to EOM or LOM samples (Fig. 3b, Additional file 1: Figure S4B). Due to the extremely low coverage of the clinical bulk sample, the read abundance variation was much higher than all other samples (Fig. 3b, Additional file 1: Figure S4B).

We then calculated the mean coefficient of variation (CV) for read abundance in the different sample types (Table 3, Fig. 3c, and Additional file 2: Table S11). Following normalization for coverage, the mean CV from the EOM/LOM samples was closer to the CV of the bulk sample than ENM/LNM samples (89/79% versus 22% versus 147/111%, respectively). Once again, the limited standard deviation in these measurements indicates that CV differences represent alterations of read uniformity in each sample type (Table 3, Additional file 2: Table S12). In support of improved uniformity with optimized MALBAC, the CV value of COM samples was similar to EOM and LOM samples (Table 3, Fig. 3c).

**Table 3:** Coefficient variation of normalized read abundance in each sample type

Sample name	Mean coefficient of variation (CV, %)	SD
*Dd2* bulk (1)	22	–
ENM (1)	147	–
EOM (13)	89	4
LNM (1)	111	–
LOM (10)	79	2
COM (2)	87	12
Clinical bulk (1)	472	–

*SD* standard deviation

### Optimized MALBAC exhibits reproducible coverage of single-cell genomes

**Fig. 4:**
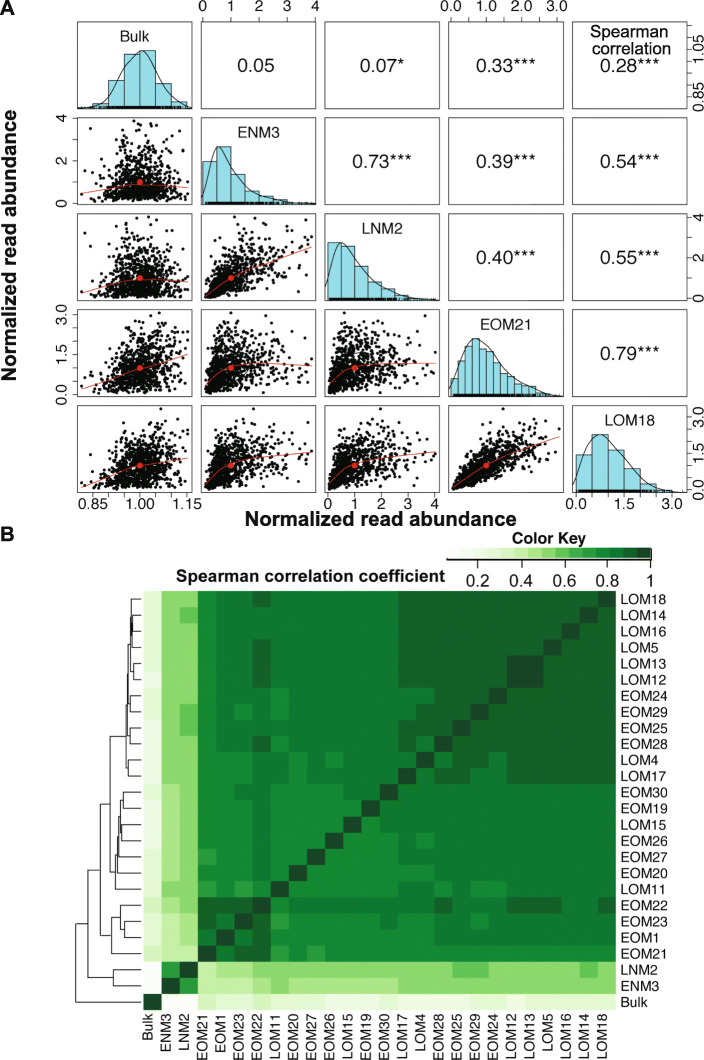
Correlations show reproducibility of amplification pattern by optimized MALBAC. **a** Paired panels for 5 × 5 matrices represent Spearman correlation, histogram, and pairwise scatterplot among the normalized read abundance of the *Dd2* bulk, ENM, LNM, and one of each EOM and LOM samples. Outlier bins were removed prior to this analysis (see “Methods” for outlier identification). The Spearman correlation coefficients of each pair are listed above the diagonal, and stars indicate the *p* value at levels of 0.1 (no star), 0.05 (*), 0.01 (**), and 0.001 (***). The histograms on the diagonal show the distribution of normalized read abundance in each sample. The bivariate scatter plots, below the diagonal, depict the fitted line through locally smoothed regression and correlation ellipses (an ellipse around the mean with the axis length reflecting one standard deviation of the *x* and *y* variables). **b** Spearman correlation coefficients between sequenced samples. The hierarchical clustering heatmap was generated using Spearman correlation coefficients of normalized read abundance. The color scale indicates the degree of correlation (white, correlation = 0; green, correlation > 0)

To better assess the amplification patterns across the genomes, we compared the distribution of binned normalized reads from single-cell samples to the bulk control using a correlation test (as performed in other single-cell studies [30, 94]). This analysis revealed that amplification patterns of optimized EOM and LOM samples were slightly correlated with the bulk control (mean Spearman correlation coefficient of 0.27 and 0.25, respectively, Additional file 2: Table S13), while the non-optimized samples were not correlated (ENM 0.05 and LNM 0.07) (Fig. 4a).

To quantify the reproducibility of read distribution between single-cell samples amplified by MALBAC, we compared Spearman correlation coefficients. The read abundance across all single-cell samples was highly correlated; two individual EOM or LOM samples had a mean correlation coefficient of 0.83 and 0.88 respectively (Fig. 4b). When we expanded our analysis to calculate the correlation of binned normalized reads between all 26 sequenced samples (Additional file 2: Table S13) and hierarchically clustered the Spearman correlation coefficient matrix between these samples, all 23 optimized single-cell samples (EOM and LOM) clustered with a mean Spearman correlation coefficient of 0.84 (Fig. 4b). In addition, the two COM samples were correlated with each other (Spearman correlation coefficient of 0.84) (Additional file 1: Figure S4C). This correlation indicated high reproducibility of normalized read distribution across the genomes that were amplified by optimized MALBAC. Within the large cluster, two LOM samples (LOM12 and LOM13) displayed the highest correlation (0.94, Fig. 4b).

### Reproducible coverage with lower variation is the main benefit of MALBAC over MDA-based amplification of single-cell genomes

We compared our data to that from a MDA-based study because this is the only other method that has been used to amplify single *Plasmodium* genomes ([16], Additional file 1: Figure S6). This study sorted individual infected erythrocytes with high (H), medium (M), and low (L) DNA content corresponding to late-, mid-, and early-stage parasites, applied MDA-based WGA to single erythrocytes, and sequenced the DNA products. The authors measured a similar amplification success rate in early (L)-stage samples as our study (MDA: 50% by DNA yield, MALBAC: 43% by DNA yield) yet slightly improved success rates for late (H)-stage samples (MDA: 100%, MALBAC: 90%, Additional file 2: Tables S8 and S9). In light of experimental differences between the two studies (Additional file 2: Table S14), we analyzed data from the twelve MDA samples using our exact analysis pipeline and parameters (six MDA-H and three of each MDA-M and MDA-L samples) and confined our comparison of the data to a few metrics: (1) coefficient of variation of read abundance, (2) coverage breadth, and (3) correlation between samples (see below).

MDA is known to produce artifacts that impair CNV detection [29, 52, 56, 95, 96]. While MALBAC-amplified genomes exhibited a consistent amplification pattern (Additional file 1: Figure S3A and S3B), the MDA-amplified genomes showed more variation across cells (Additional file 1: Figure S6A). We also detected higher variation in normalized read abundance in the MDA-H samples (compared to MDA-L and MDA-M samples, Additional file 1: Figure S6B), which was not consistent with the report that the MDA method amplifies high DNA content better than parasites with lower DNA content [16]. Even though the bulk DNA controls used in both studies showed similar CVs (24% versus 22%), the MDA-amplified samples displayed a higher CV than MALBAC-amplified single-cell samples regardless of the parasite stage (a mean of 186% versus 85%, respectively, Table 3, Additional file 2: Tables S11 and S15). As expected based on MALBAC’s limited coverage of intergenic regions (Table 2), MDA-amplified samples displayed a higher coverage breadth cross the genome, especially in the intergenic regions (Additional file 2: Table S16). Additionally, the correlation between MDA-amplified cells (mean correlation coefficient: 0.20; Additional file 2: Table S17, Additional file 1: Figure S6D) was much lower than that between our optimized MALBAC-amplified cells (mean correlation coefficient: 0.84; Additional file 2: Table S13, Fig. 4b); this finding confirms prior observations that MDA exhibits a more random amplification pattern than MALBAC [97].

### Copy number variation analysis is achievable in MALBAC-amplified single-cell genomes

To detect CNVs with confidence, we employed both discordant/split read detection and read-depth based methods with strict parameters. We used LUMPY to detect paired reads that span CNV breakpoints or have unexpected distances/orientations (requiring a minimum of 2 supporting reads). We also used a single-cell CNV analysis tool, Ginkgo, to segment the genome based on read depth across bins of multiple sizes and determine copy number of segments (requiring a minimum of 5 consecutive bins). We regarded the CNVs detected by the two methods as the same if one CNV overlapped with at least half of the other CNV and vice versa (50% reciprocal overlap). Using this approach, we first identified a “true set” of CNVs from the bulk *Dd2* DNA sample (Table 4, 3 CNVs on 3 different chromosomes). One of the true CNVs was identified previously in this parasite line (the large *Pfmdr1* amplification on chromosome 5, [66]); another true CNV occurs in an area of the genome that is reported to commonly rearrange in laboratory parasites ([98], the *Pf11-1* amplification of chromosome 10).

**Table 4:** True CNVs detected in the *Dd2* bulk genome

Name	Chr.	Start Pos.	Size (bp)	Type	Support read*		Start Pos.	Size (bp)	Copy number detected by Ginkgo** in different bin sizes				Mappability^
					Discordant read	Split read			1 kb	5 kb	8 kb	10 kb	
*Pfmdr1*	5	888316	81,935	DUP	53	0	888000	82,000	2	2	Nd	Nd	1
*Pf11-1*	10	1524527	18,472	DUP	29	1	1520000	28,000	4	5	N/A	N/A	0.86
*Pf332*	11	1956623	8719	DUP	0	8	1953000	13,000	4	N/A	N/A	N/A	0.92

*Detected by LUMPY based on discordant/split read detection, minimum number of supporting reads is 2

**For Ginkgo analysis, the minimum bin number of segmentation is 5

^For comparison, the mean mappability of the core genome is 0.99 and the mean mappability telomere/subtelomere regions including *var* gene clusters is 0.65

*DUP* duplication, *N/A* not applicable because the target CNVs will not be detected as the bin size (≥ 5× bin size) is larger than the size of the target CNVs, *Nd* not detected in the specified bin size

With a set of true CNVs in hand, we assessed our ability to identify them in the single-cell samples amplified by MALBAC and explored parameters that impacted their detection. As expected, each CNV detection method exhibited differences in the ability to identify true CNVs, which is likely due to a number of factors including CNV size, genomic neighborhood, and sequencing depth [99]. For example, using discordant/split read analysis, we were able to readily identify the *Pf11-1* amplification in the majority of samples (21 of 25 samples, Additional file 2: Table S18). This method was less successful in identifying the *Pfmdr1* amplification (only 3 optimized MALBAC samples in total, Additional file 2: Table S18). For read-depth analysis, the success of true CNV detection was heavily dependent on the bin size (Additional file 2: Table S18). If we selected the lowest bin size (1 kb) in which it was possible to detect the smallest of the true CNVs (13 kb), we were able to readily identify the *Pfmdr1* amplification in all samples (Additional file 2: Table S18). As we increased the bin size, the number samples with *Pfmdr1* detection decreased, only optimized MALBAC samples were represented, and the copy number estimate in single cells approached the bulk control (Additional file 2: Tables S7 and S18). The other two true CNVs were only detected at the 1 kb bin size in a minority of samples (6 total, Additional file 2: Table S18).

When we assessed true CNVs that overlapped between the two methods, we were able to improve the precision and sensitivity of CNV detection in five single-cell samples (Table S19) and detect at least one CNV in each (3 EOM and 2 LOM samples out of 25 total cells, Table 5). Notably, in one sample, EOM 23, the *Pfmdr1* amplification was detected in bin sizes of up to 10 kb at a copy number similar to the bulk control (Table 5). Besides the CNVs conserved with the bulk sample, we also detected unique CNVs that were only identified in the single-cell samples. In general, the CNVs detected by both discordant/split read and read depth analyses were spread across all chromosomes except chromosome 9, predominantly confined to optimized MALBAC samples, and were only detected at 1 kb read depth bin sizes (Additional file 2: Table S20).

**Table 5:** True CNVs detected in single-cell samples

Sample name	CNV name	Start position	Size (bp)	Supporting reads		Start position	Size (bp)	Copy number detected by Ginkgo in different bin sizes			
				Discordant read	Split read			1 kb	5 kb	8 kb	10 kb
LOM 5	*Pfmdr1*	891390	34,069	0	2	907000	28,000	9	Nd	N/A	N/A
LOM 16	*Pf11-1*	1542335	3836	0	3	1543000	5000	3	N/A	N/A	N/A
EOM 23	*Pfmdr1*	889899	79,890	3	3	888000	82,000	4	6	5	5
EOM 26	*Pf11-1*	1542335	3836	0	5	1543000	5000	4	N/A	N/A	N/A
EOM 29	*Pf11-1*	1539158	5639	4	0	1541000	7000	3	N/A	N/A	N/A

*N/A* indicates the target CNVs will not be detected as the bin size (≥ 5 bin size) is larger than the size of the target CNVs. *Nd* not detected in the specified bin size

### High-quality SNPs are detected in MALBAC-amplified single-cell genomes

Firstly, to understand the accuracy of SNP detection in MALBAC-amplified genomes, we estimated the precision and sensitivity of SNP detection in single cells by treating those from the *Dd2* bulk sample as standard SNP set. We performed this analysis with increasing stringency levels (VQSLOD score > 0; VQSLOD score > 6; VQSLOD score > 6 with read depth > 10, Table 6) in order to calibrate with previous SNP studies and evaluate the impact of read depth on SNP identification. In the *Dd2* bulk sample, 18,369 SNPs were detected with VQSLOD score > 0, while 13,168 SNPs were detected with VQSLOD score > 6 and read depth > 10; the later number is more consistent with the number of SNPs identified in previous studies of *Dd2 P. falciparum* [100]. Similarly, as we increased the stringency level, fewer SNPs were detected for each single-cell sample and sensitivity decreased, indicating increased false negatives for SNP detection. The precision of SNP detection, however, increased from 65% (VQSLOD score > 0) to 92% (VQSLOD score > 6) and 99% (VQSLOD score > 6/read depth > 10) in EOM samples; the same trend was observed for LOM samples (Table 6). The best balance of precision and sensitivity for SNP detection in single cells was achieved at the level of VQSLOD score > 6. Even though the sensitivity for SNP detection is only 46% (EOMs) and 47% (LOMs) in individual single cells at this stringency level, we observed up to 72% sensitivity when we pooled optimized single-cell samples (13 EOMs and 10 LOMs, Figure S7A).

**Table 6:** SNP detection in sequenced samples

Variant filtering conditions	Sample name	Number of SNPs	Precision	Sensitivity
VQSLOD > 0	EOM (13)	12,734	65.25%	45.09%
	LOM (10)	12,730	67.16%	46.40%
	ENM (1)	2269	84.93%	10.49%
	LNM (1)	9235	67.29%	33.83%
	*Dd2* bulk (1)	18,369	–	–
	COM (2)	8851	31.55%	15.22%
VQSLOD > 6	EOM (13)	6917	91.75%	46.22%
	LOM (10)	6990	92.40%	47.05%
	ENM (1)	1375	96.58%	9.68%
	LNM (1)	4902	95.17%	33.99%
	*Dd2* bulk (1)	13,725	–	–
	COM (2)	3162	66.69%	15.37%
VQSLOD > 6, read depth > 10	EOM (13)	3937	99.48%	29.74%
	LOM (10)	4360	99.41%	32.92%
	ENM (1)	252	97.62%	1.87%
	LNM (1)	2575	98.87%	19.33%
	*Dd2* bulk (1)	13,168	–	–
	COM (2)	1021	79.28%	6.13%

*Precision and sensitivity are calculated by using SNPs detected in *Dd2* bulk sample as the standard SNP set

We also evaluated the detection of 16 known drug resistance SNPs from the *Dd2* bulk sample (Additional file 2: Table S21). When we pooled all single-cell samples (EOMs and LOMs), we detected 13 of the 16 resistance SNPs (Additional file 2: Table S22); the 3 remaining SNPs were not identified due to a lack of coverage at these sites in single-cell genomes (Additional File 3: SNPs detected in all samples). As expected, the sensitivity of SNP detection was much lower in non-*Dd2* patient-isolated COM samples (15.37%, VQSLOD score > 6) when compared to that in the *Dd2*-derived EOM samples (46.22%).

Since the *Dd2* parasites that we used in this study were not recently cloned, there is a possibility of detecting novel SNPs that have arisen in the population over time in laboratory culture [17]. After removing any mixed allele calls and applying the highest stringency level (VQSLOD score > 6, read depth > 10), we identified 124 novel SNPs in the single-cell samples that were not present in the *Dd2* bulk sample (Additional file 4: Single cell novel SNPs). These loci affected 226 genes on all 14 chromosomes of the parasite genome (Additional file 2: Table S23), representing genes involved in the biosynthesis of antibiotics, Ac/N-end rule pathway, purine metabolism, thiamine metabolism, and aminoacyl-tRNA biosynthesis (Additional file 2: Table S24).

## Discussion

This study is the first to optimize the standard MALBAC protocol for single-cell sequencing of a genome with extreme GC content (*P. falciparum:* 19.4% GC). We showed that this optimized method can reliably amplify early-stage parasite genomes, which contain < 30 fg of DNA per sample. Single-cell experiments are innately very sensitive to contaminating DNA from other organisms and we detected a lower proportion of human and bacteria DNA in MALBAC-amplified samples, which impacted overall coverage of the *P. falciparum* genome. Furthermore, we showed that this method exhibited reduced GC bias to impact the breadth and uniformity of genome amplification. Finally, with these single-cell genomes, we were able to explore the detection of CNVs and SNPs to study parasite-to-parasite heterogeneity.

### MALBAC volume and cycles

MALBAC amplification has been used in studies of human cells, where each diploid genome harbors ~ 7 pg of DNA [43, 45]. In this study, we used the MALBAC method to successfully amplify femtogram levels of DNA from single *P. falciparum* parasites. Reducing the total reaction volume (from 50 to 20 μl) and increasing the number of amplification cycles (pre-amplification: from 5 to 18–19; exponential: from 15 to 17) likely contributed significantly to this increased sensitivity. Both modifications were important. Initially the lower sample volume reduced the overall DNA yield, and this was reversed using increased amplification cycles. These modifications provide additional benefits including reduced contaminating reads and experimental costs. Importantly, these simple steps can be applied to the MALBAC amplification of small genomes or genomes with skewed GC content from other organisms such as bacteria [101]. For example, studies of *Mycoplasma capricolum* (GC-poor) [102]*, Rickettsia prowasekii* (GC-poor) [103], and *Borrelia burgdorferi* (GC-poor) [104], *Entamoeba histolytica* (GC-poor) [105], and *Micrococcus luteus* (GC-rich) [106] could be improved using this method.

### Primers and coverage bias

The modification of the primer was essential for the successful amplification of the AT-rich *P. falciparum* genome. This change was meant to prevent the preferential amplification of GC-rich sequences as observed for human and rat single-cell genomes [30, 46]. We achieved a coverage breadth across *P. falciparum* genic regions (a mean of 21.7% GC content) of as high as ~ 80% (Table 2) by specifically altering the base content of the degenerate 5-mer of MALBAC pre-amplification primer from 50 to 20% GC content. The initial priming step is crucial for whole-genome amplification and controlling this step can limit amplification bias [107]. Indeed, 5-mers with ~ 20% GC content across the *P. falciparum* genome are 2- and 6-fold more common than those with 40% and 60% GC content, respectively (Additional file 2: Table S1). This difference indicated that annealing of the optimized MALBAC primer based on the degenerate bases was more specific for the parasite’s genome than the standard MALBAC primer. Interestingly, during this study, we observed a preferential amplification of genic over intergenic regions (Table 2), which may be explained by lower percentage of 5-mers with 20% GC content in intergenic regions than in genic regions (Additional file 2: Table S1). Furthermore, when we searched for reads that contained the MALBAC common sequence (see “Methods” and Additional file 2: Table S5) to identify WGA binding sites across the *P. falciparum* genome, we found that binding sites were predominantly located in the genic regions (Additional file 2: Table S5); this result indicated that there was an issue with primer annealing in intergenic regions, which may be caused by a high predicted rate of DNA secondary structure formation across these regions of the *P. falciparum* genome [53]. The polymerase used in the MALBAC linear amplification steps (*Bst* large fragment) exhibits strand displacement activity, which presumably allows resolution of secondary structure [108, 109]. However, a longer extension time may be required for amplification of repetitive DNA sequence, either during linear or exponential steps.

### Parasite and contaminating genomes

The standard MALBAC method was previously reported to display the most favorable ratio of parasite DNA amplification over human DNA when compared to other common WGA methods [110]. Out steps to optimize MALBAC (reduced volume and increased cycle numbers) not only enhanced the amplification of the small parasite genome, but also likely improved the sensitivity to amplify contaminating non-parasite DNA. Nevertheless, in many samples, optimized MALBAC yielded lower proportions of contaminating DNA than the bulk sample (Fig. 2a). We speculate that this effect was once again due to our modification of the GC content of the degenerate bases of the primer; this alteration limited the preferential amplification of contaminating DNA with higher GC content (observed during standard MALBAC), thus improving the representation of parasite DNA in the overall WGA product.

The major contaminating DNA source that we detected in our samples was from humans (Fig. 2a). This was not surprising given that, in our experimental system, the culture and host environments are rich in human DNA [92, 93, 111]. It is also possible that human DNA was introduced during the single-cell isolation or WGA steps [56]. The former situation is a larger issue for clinical parasite isolates due to the abundance of white blood cells that contribute to extracellular DNA when they decay outside of the host [112]. Indeed, we observed more human DNA in clinical bulk and single-cell samples (an increase of ~ 11-fold over laboratory-derived *Dd2* bulk and single-cell samples, respectively). The massive level of contamination in the clinical bulk sample and limited coverage of the parasite genome (0.3%) was exacerbated by (1) the omission of a stringent leukodepletion step that is routinely employed to limit host cell contamination [13, 113, 114] and (2) the lower overall sequencing output of that particular run (Additional file 2: Table S4).

The second most common source of contaminating DNA was bacteria (Fig. 2a). WGA approaches are known to occasionally amplify residual bacterial DNA associated with commercial polymerases [115–118] or other reagents [119–121]. Since this contaminant was absent in the bulk DNA control and increased in early-stage parasite samples (representing an average of 0.8% of EOM reads compared to 0.2% for LOM samples), bacterial DNA may also have been introduced during single parasite isolation and WGA steps. While we took precautions to limit this occurrence (see “Methods”), minimizing the reaction volume could further reduce this source of contamination.

### Early- and late-stage parasites

Depending on when a novel CNV or SNP arises (i.e., early or late in replication), each parasite stage holds advantages for its detection. If the mutation arises in the first round of replication and is copied into most of the genomes of a late-stage parasite, having multiple genomes will be advantageous for detection. If the mutation arises later in replication, it will be present in few of the genomes; therefore, averaging across the genomes, as with bulk analysis, will limit its detection. Since only one haploid genome is present in an early-stage parasite, the sensitivity for detecting rare/unique CNVs/SNPs within parasite populations is favored.

For this reason, staging of parasites in this study was important. We performed stage-specific enrichment before single-cell isolation and confirmed that the majority of parasites were the desired stage using flow cytometry (see “Methods,” Additional file 1: Figure S1, 97% for early-stage enrichment and 74% for late-stage enrichment). Furthermore, during selection of cells by microscopy (before automated collection by the Cell Raft instrument), we confirmed the expected fluorescence intensities for each stage; early-stage parasites had a significantly smaller genome and mitochondrion size compared to late state (as in Fig. 1b). However, differences in preparation of samples may have impacted our parasite stage comparisons. While all late-stage samples were isolated, lysed, and amplified in the same batch under the same conditions, early-stage samples were processed in three separate batches (Additional file 2: Table S11). Despite conserved methods and good concordance in CV between all samples (Additional file 2: Table S11), minor differences could have contributed to variations in the amplification steps.

Differences in genome analysis results from optimized MALBAC samples provided further confidence that the parasites were of the expected stage. Firstly, late-stage parasites showed a higher WGA success rate than early-stage parasites (90% versus 43%, Additional file 2: Table S9). This result was explained simply by the presence of extra genomes in the late-stage samples (~ 16n versus 1n) and was consistent with a previous study that used MDA-based amplification methods [16]. Late-stage parasites also displayed better uniformity of read abundance (Table 3), indicating less amplification bias because fewer regions were missed when more genomes were present. Additionally, there were fewer overall contaminating reads found in late-stage parasites than early-stage parasites (5.1% versus 8.6%). Once again, this was likely due to a higher ratio of parasite DNA to contaminating DNA in the late-stage samples.

Despite these differences, we observed similar coverage breadth and Spearman correlation coefficients of read abundance for both early- and late-stage MALBAC-amplified parasites (Table 2 and Additional file 2: Table S13). This was contrary to the MDA study in single *P. falciparum* parasites that found a higher breadth of genome coverage from the late-stage parasites [16]. Our findings confirm that the pattern of amplification across the genome is determined by the binding of the optimized MALBAC primers and not the parasite developmental form.

### CNV analysis and related considerations

Sequencing at very high depth improves the detection of low frequency CNVs in bulk samples, but the sensitivity is limited to large-scale CNVs present in > 5% cells [33, 45, 122]. Other analysis methods that rely on the detection of reads that span CNV junctions (i.e., split reads or discordant reads) have improved the sensitivity and specificity of CNV detection [123], but continue to struggle with minor allele detection. For single-cell analysis, the high level of MALBAC amplification reproducibility (i.e., the same regions are over- and under-amplified across multiple genomes), that we and others have observed, is especially advantageous for CNV detection. This is because amplification bias can be normalized across cells, as has been successfully performed for human cells [45, 124]. Unfortunately, cross-sample normalization was not possible in our study due to the use of a single laboratory parasite line that includes known CNVs (*Dd2*). Instead, as described below, we combined a read-depth based tool (Ginkgo [82]) with a split/discordant read-based method (LUMPY [81]) to improve the accuracy of CNV detection (as in [99]).

We observed a large number of raw CNVs detected in single-cell genomes by each individual method (Additional file 2: Table S6-LUMPY and Table S7-Ginkgo) and the precision and sensitivity of each method was low (Additional file 2: Table S19). These initial results may be explained by a number of possibilities, including those that are both biological in nature as well as artifacts of our analysis methods. From a biological perspective, these calls can represent large CNVs that are known to exist in the bulk sample (i.e., *Pfmdr1* and *Pf11.1*, Table 5) as well as the abundance of small CNVs [22] that may be present in a minor part of the population (unique CNVs, Additional file 2: Table S20). Because prior *P. falciparum* CNV analyses were confined to bulk DNA sequencing, our view of minor variants in parasite populations is limited. The recent discovery of *P. falciparum* extrachromosomal DNA that is derived from regions of the genome that harbor CNVs [125] suggests that there are cellular pathways that could contribute to cell-to-cell variations in CNV boundaries and dynamics (i.e., perhaps through the excision and reintegration of extrachromosomal DNA). While the differences in start position of true CNVs from single-cell genomes (Tables 4 and 5) could represent true minor variants, they could also be due to analysis artifacts that contribute to excess CNV calls and inaccuracies in estimating boundaries. For example, raw LUMPY results exhibit redundancy due to slightly varied boundaries and sizes of the same CNV. Additionally, parameters of read-depth-based approaches like Ginkgo (i.e., bin sizes and the requirements for consecutive bins) can alter CNV calling; 1-kb bins may heavily reflect coverage variation in the genome and have a high level of false positives while larger bin sizes may miss smaller CNVs. In an effort to limit false positives and these uninformative variations, we combined approaches by retaining calls that overlapped between the two approaches (see “Methods”). In support of this combined approach, we detected a decrease in the number of overall CNV calls and improvements in the precision in some single-cell samples (Additional file 2: Table S19).

Despite these improvements, we observed variations in the boundaries and copy numbers of the true CNVs in single-cell samples (Tables 4 and 5). For example, in *Dd2* parasites, the *Pfmdr1* CNV is ~ 82 kb but in single-cell samples, it is called as ~ 30 kb with a later starting position. This difference is most likely due to uneven coverage across these large CNVs in single-cell samples; some regions accurately reflect the CNV where others do not. Importantly, as we increased the bin size, the uniformity of read count improves (Additional file 1: Figure S5), which impacts CNV identification (i.e., the *Pfmdr1* amplification is found in fewer single-cell genomes and the copy number estimate approaches that of the bulk control, Additional file 2: Tables S7 and S18). Thus, efforts to improve the uniformity of read coverage, genome coverage breadth, and the potential for cross-sample normalization will improve our ability to accurately detect CNVs. Overall, it is notable that we can detect CNVs in some single-cell genomes (< 100 kb, Table 5) that are below the current resolution of CNV detection from single-cell genomes amplified with common WGA methods (>> 100 kb to Mb) [33, 39, 44–49]. Our CNV analysis capabilities will improve with expanded numbers and genomic diversity; the inclusion of parasite lines with different CNV profiles will greatly facilitate the removal of reproducible amplification bias and increase the detection of conserved and unique CNVs of all sizes.

### Standard and novel SNP analysis

By combining stringent SNP filtering strategies (i.e., VQSLOD and read depth cutoffs), we increased the precision for SNP calling in single-cell samples and detected 72% of SNPs identified in the bulk sample and 13 of 16 known resistance SNPs (Table 6, Table S22, Figure S7A). We also detected a number of novel SNPs across our single-cell samples (Additional file 4: Single cell novel SNPs VCF file). On average, this is ~ 13 SNPs per genome, since many of the 124 novel SNPs are shared among genomes (46 shared SNPs). We are not able to  directly compare this rate to that estimated from other studies [4, 17, 89, 126, 127] because our bulk sample was not cloned prior to single-cell isolation and overall culturing days are unknown. Although we take precautions to limit divergence (i.e., parasite lines are only grown for limited amounts of time and periodically cloned), we do not know the complete life history of the *Dd2* line because it was acquired from a general repository. However, when we assessed SNPs from multiple *Dd2* lines using the current pipeline (see “Methods,” VQSLOD> 6), we identified 146 SNPs in the short reads used to generate the *Dd2* reference genome [80] that were not present in our bulk sample, indicating some divergence occurs in samples that are independently propagated.

### Limitations, scope, and future efforts

One limitation in our comparison between standard and optimized MALBAC-amplified samples was that we only sequenced a single standard MALBAC sample from each parasite stage. However, during our studies we evaluated a total of 7 independent non-optimized samples using ddPCR (3 ENM and 4 LNM) and detected multiple instances of allelic dropout and heavy skewing of the copy number of a known CNV (Table 1 and Additional file 2: Table S10). These results indicated extreme bias coverage and high levels of contaminating DNA, which made sequencing of these samples futile. Nevertheless, evaluating specific genes is not equivalent to sequencing a whole genome. Thus, while we have adapted MALBAC for amplifying single *P. falciparum* parasite genomes, further studies are required to rigorously evaluate the differences between standard and optimized MALBAC.

A second limitation of our study was our inability to directly compare MALBAC results to those produced using MDA. Our studies specifically sought to adapt MALBAC for amplification of the *Plasmodium* genome; therefore, we did not perform MDA on our samples in parallel. However, in order to gain some insight into the performance of the two WGA methods on the *P. falciparum* genome, we performed limited comparisons with data from a previous MDA-based study (Fig. 4b, Additional file 1: Figure S3 versus S6; Table 2 versus Additional file 2: Table S16; Table S11 versus S15; Table S13 versus S17). Direct comparisons were constrained by the use of distinct parasite lines (*HB3* vs *Dd2*) and single-cell preparation pipelines but the results emphasized the strengths and weaknesses of each method. While MDA is known to exhibit lower single-nucleotide amplification error and acquired overall higher genome coverage in *P. falciparum* genome [16] (Additional file 2: Table S16), it is not suitable for CNV detection [52]. MALBAC, on the other hand, can provide high-quality SNP identification following strict filtering steps ([97] and Table 6), and the reproducible amplification pattern (Fig. 4b) can be beneficial for both CNV and SNP detection (see below).

Another limitation is related to the lack of MALBAC coverage across certain genomic regions (~ 40% of the overall genome, ~ 70% of the intergenic regions, Table 2), which impacts the detection of genetic variations in these locations. Low read depth resulted in the failure to detect SNPs (Figure S7B) and variation of coverage leads to inaccuracies in the size and boundaries of CNVs (see “CNV analysis and related considerations”). However, the reproducible pattern of genome coverage by MALBAC provides some advantages. First, as mentioned above, we can exploit this feature to normalize across diverse samples to minimize noise and improve CNV detection; any improvements in the coverage of intergenic regions and uniformity will also impact CNV identification through increased detection of discordant/split reads and more accurate read-depth calling. Second, the consistent coverage pattern allows us to predict a defined set of SNPs that can be consistently detected across pooled single-cell samples with a given coverage level.

Finally, we specifically recognize the limitations of our CNV analysis pipeline. First, we confined our assessments to duplications and deletions (Additional file 2: Table S19) but have not evaluated other types of structural variations that may also be important for adaptation. Second, we acknowledge that our CNV analysis on single-cell genomes is not yet robust (see “CNV analysis and related considerations” above). We also recognize that there is a tradeoff between sensitivity and precision during CNV analysis; accepting the possibility of false positives allows maximal sensitivity to detect novel CNVs. Ultimately, the benefit of single-cell genomics is the discovery of minor variants that provide insight into the dynamics of adaptation. While we would not consider individual CNVs identified in our current analysis to be particularly informative, studies assessing relative CNV levels (under condition 1 vs 2) would likely yield informative results using the current methods. To achieve consistent and robust CNV calling, we require a combination of improvements in both amplification methods and analysis tools (as proposed above). This study can be used as a springboard for such advancements.

## Conclusions

It is notable that we can successfully amplify a small, base-skewed genome and detect genetic variations on a single-cell level. Our modifications of reaction volume, cycle number, and GC content of degenerate primers will expand the use of MALBAC-based approaches to organisms not previously accessible because of small genome size or skewed base content. Furthermore, these changes can reduce amplification of undesired contaminating genomes in a sample. The reproducible nature of this WGA method, combined with new genome analysis tools, will reduce the effect of amplification bias when conducting large-scale single-cell analysis and enhance our ability to explore genetic heterogeneity in the form of both SNPs and CNVs. Thus, we expect this approach to broadly improve study of mechanisms of genetic adaptation in a variety of organisms.

## Supplementary Information

**Additional file 1: Figure S1.** Confirmation of staging for enriched parasite samples. **Figure S2.** Bioinformatic analysis of sequencing reads. A. Whole genome sequencing analysis and alignment. **Figure S3.** Uniformity of read abundance across the whole genome of all EOM and LOM samples. **Figure S4.** Uniformity of read abundance across the whole genome and correlation analysis for clinical samples. **Figure S5.** Distribution of normalized read counts in various bins sizes. **Figure S6.** Uniformity of coverage and correlation analysis in MDA-amplified single cell samples. **Figure S7.** Sensitivity of SNP detection of pooled single cells and association between SNP calls and read depth.

**Additional file 2: Table S1.** GC content of 5 base windows in the *P. falciparum* genome. **Table S2.** Primer and probe design for droplet digital PCR. **Table S3.** Correlation between ddPCR gene copy concentration and sequencing depth of ddPCR target. **Table S4.** Overall sequencing read output and percentage of aligned reads to *P. falciparum* genome. **Table S5.** Proportion of reads that contain the MALBAC primer common sequence and their alignment to specific genomic regions. **Table S6.** CNVs detected by LUMPY in all samples (single cell and *Dd2* bulk). **Table S7.** CNVs detected by Ginkgo in all samples (single cell and *Dd2* bulk). **Table S8.** The number of single cell samples processed at each analysis step. **Table S9.** DNA yield after MALBAC amplification. **Table S10.** Primary data from ddPCR detection: calculation of uniformity score and *Pfmdr1* copy number. **Table S11.** Coefficient of variation of normalized read abundance in each sequenced sample. **Table S12.** The equality of CVs in normalized read abundance for sequenced samples. **Table S13.** Spearman correlation coefficient of all sequenced samples. **Table S14.** Comparison between experimental conditions of MALBAC and MDA-amplified single cell samples. **Table S15.** The coefficient of variation of normalized read abundance in MDA amplified samples. **Table S16.** Coverage comparison after downsampling to 300,000 reads. **Table S17.** Spearman correlation coefficient of MDA amplified samples. **Table S18.** Detection of true CNVs in single cell genomes by discordant/split read or read depth analysis. **Table S19.** Precision and sensitivity of CNV detection. **Table S20.** Single cell CNVs detected by both discordant/split read and read depth analysis (excluding true CNVs presented in Table 5). **Table S21.** Detection of known SNPs in resistant genes in *Dd2* bulk sample. **Table S22.** Detection of known SNPs in resistant genes in single cell samples. **Table S23.** Subpopulation SNPs detected in single cell samples. **Table S24.** Pathways of genes affected by novel SNPs detected in single cell samples.

**Additional file 3.** SNPs detected in all samples. Excel file of SNPs detected in all samples with gene name annotation (VQSLOD > 6).

**Additional file 4.** Single cell novel SNPs. Excel file of Single cell novel SNPs with gene name annotation (VQSLOD > 6, DP > 10).

## Data Availability

The data analysis pipeline is publicly available at https://github.com/Pfal-analysis/Single-cell-sequencing-data [128]. The raw sequence files generated and analyzed during the current study are available in the Sequence Read Archive (SRA) under the BioProject ID PRJNA607987, BioSamples SAMN14159290-SAMN14159318, and can be accessed here: https://www.ncbi.nlm.nih.gov/bioproject/PRJNA607987/ [129]. The datasets for the uniformity and reproducibility analysis of MDA-based amplification on parasite DNA from single infected erythrocytes were obtained from Trevino et al. [16], SRA accession PRJNA385321.

## Abbreviations

MALBAC: Multiple annealing and looping-based amplification cycles
CNVs: Copy number variations
SNPs: Single-nucleotide polymorphisms
WGA: Whole-genome amplification
MDA: Multiple displacement amplification
*Pfmdr1*: *Plasmodium falciparum multidrug resistance 1*
ddPCR: Droplet digital PCR
NA: Not applicable
SD: Standard deviation
EOM: Early-stage single parasites amplified by optimized MALBAC
LOM: Late-stage single parasites amplified by optimized MALBAC
COM: Clinical single parasites amplified by optimized MALBAC
ENM: Early-stage single parasites amplified by non-optimized MALBAC
LNM: Late-stage single parasites amplified by non-optimized MALBAC
IQR: Interquartile range
CV: Coefficient of variation
SLOPE: Streptolysin-O Percoll
VQSLOD: Variant quality score log-odds

## References

[1] Rich SM, Leendertz FH, Xu G, LeBreton M, Djoko CF, Aminake MN (2009). The origin of malignant malaria. Proc Natl Acad Sci U S A..

[2] Matthews H, Duffy CW, Merrick CJ (2018). Checks and balances? DNA replication and the cell cycle in Plasmodium. Parasites Vectors.

[3] Blasco B, Leroy D, Fidock DA (2017). Antimalarial drug resistance: linking *Plasmodium falciparum* parasite biology to the clinic. Nature medicine..

[4] Bopp SER, Manary MJ, Bright AT, Johnston GL, Dharia NV, Luna FL (2013). Mitotic evolution of *Plasmodium falciparum* shows a stable core genome but recombination in antigen families. PLoS Genet.

[5] Cheeseman IH, Gomez-Escobar N, Carret CK, Ivens A, Stewart LB, Tetteh KKA (2009). Gene copy number variation throughout the *Plasmodium falciparum* genome. BMC Genomics..

[6] Guler JL, Freeman DL, Ahyong V, Patrapuvich R, White J, Gujjar R (2013). Asexual populations of the human malaria parasite, *Plasmodium falciparum*, use a two-step genomic strategy to acquire accurate, beneficial DNA amplifications. PLoS Pathogens..

[7] Kidgell C, Volkman SK, Daily J, Borevitz JO, Plouffe D, Zhou Y (2006). A systematic map of genetic variation in *Plasmodium falciparum*. PLoS Pathog.

[8] Nair S, Miller B, Barends M, Jaidee A, Patel J, Mayxay M (2008). Adaptive copy number evolution in malaria parasites. PLOS Genet.

[9] Ribacke U, Mok BW, Wirta V, Normark J, Lundeberg J, Kironde F (2007). Genome wide gene amplifications and deletions in *Plasmodium falciparum*. Mol Biochem Parasitol.

[10] Hyde JE (2007). Drug-resistant malaria - an insight. FEBS J..

[11] Conway DJ (2007). Molecular epidemiology of malaria. Clin Microbiol Rev..

[12] Menard D, Dondorp A (2017). Antimalarial drug resistance: a threat to malaria elimination. Cold Spring Harb Perspect Med..

[13] Venkatesan M, Amaratunga C, Campino S, Auburn S, Koch O, Lim P (2012). Using CF11 cellulose columns to inexpensively and effectively remove human DNA from *Plasmodium falciparum*-infected whole blood samples. Malar J..

[14] Ibrahim A, Diez Benavente E, Nolder D, Proux S, Higgins M, Muwanguzi J (2020). Selective whole genome amplification of Plasmodium malariae DNA from clinical samples reveals insights into population structure. Sci Rep.

[15] Melnikov A, Galinsky K, Rogov P, Fennell T, Van Tyne D, Russ C (2011). Hybrid selection for sequencing pathogen genomes from clinical samples. Genome Biol.

[16] Trevino SG, Nkhoma SC, Nair S, Daniel BJ, Moncada K, Khoswe S (2017). High-resolution single-cell sequencing of malaria parasites. Genome Biol Evol..

[17] Jett C, Dia A, Cheeseman IH. Rapid emergence of clonal interference during malaria parasite cultivation. bioRxiv. 2020:2020.03.04.977165.

[18] Lähnemann D, Köster J, Szczurek E, McCarthy DJ, Hicks SC, Robinson MD (2020). Eleven grand challenges in single-cell data science. Genome Biol.

[19] Price RN, Uhlemann A-C, Brockman A, McGready R, Ashley E, Phaipun L (2004). Mefloquine resistance in *Plasmodium falciparum* and increased pfmdr1 gene copy number. Lancet..

[20] Ravenhall M, Benavente ED, Sutherland CJ, Baker DA, Campino S, Clark TG (2019). An analysis of large structural variation in global *Plasmodium falciparum* isolates identifies a novel duplication of the chloroquine resistance associated gene. Sci Rep..

[21] Heinberg A, Siu E, Stern C, Lawrence EA, Ferdig MT, Deitsch KW (2013). Direct evidence for the adaptive role of copy number variation on antifolate susceptibility in *Plasmodium falciparum*. Mol Microbiol.

[22] Cheeseman IH, Miller B, Tan JC, Tan A, Nair S, Nkhoma SC (2016). Population structure shapes copy number variation in malaria parasites. Mol Biol Evol.

[23] Lauer S, Avecilla G, Spealman P, Sethia G, Brandt N, Levy SF (2018). Single-cell copy number variant detection reveals the dynamics and diversity of adaptation. PLoS Biol..

[24] Wang R, Lin D-Y, Jiang Y (2020). SCOPE: a normalization and copy-number estimation method for single-cell DNA sequencing. Cell Syst.

[25] Wang X, Chen H, Zhang NR (2018). DNA copy number profiling using single-cell sequencing. Brief Bioinform..

[26] Gawad C, Koh W, Quake SR (2016). Single-cell genome sequencing: current state of the science. Nat Rev Genet.

[27] Macaulay IC, Voet T (2014). Single Cell Genomics: Advances and Future Perspectives. PLoS Genet.

[28] Wang Y, Navin NE (2015). Advances and applications of single-cell sequencing technologies. Mol Cell..

[29] He F, Zhou W, Cai R, Yan T, Xu X. Systematic assessment of the performance of whole-genome amplification for SNP/CNV detection and β-thalassemia genotyping. Am J Hum Genet. 2018;63:407–16. 10.1038/s10038-018-0411-5 29440707

[30] Hou Y, Wu K, Shi X, Li F, Song L, Wu H, et al. Comparison of variations detection between whole-genome amplification methods used in single-cell resequencing. GigaScience. 2015;4. 10.1186/s13742-015-0068-3. 10.1186/s13742-015-0068-3 PMC4527218 26251698

[31] Huang L, Ma F, Chapman A, Lu S, Xie XS (2015). Single-cell whole-genome amplification and sequencing: methodology and applications. Annu Rev Genom Hum Genet..

[32] Deleye L, Tilleman L, Vander Plaetsen A-S, Cornelis S, Deforce D, Van Nieuwerburgh F (2017). Performance of four modern whole genome amplification methods for copy number variant detection in single cells. Sci Rep..

[33] Chronister WD, Burbulis IE, Wierman MB, Wolpert MJ, Haakenson MF, Smith ACB (2019). Neurons with complex karyotypes are rare in aged human neocortex. Cell Rep.

[34] Duan M, Hao J, Cui S, Worthley DL, Zhang S, Wang Z (2018). Diverse modes of clonal evolution in HBV-related hepatocellular carcinoma revealed by single-cell genome sequencing. Cell Res.

[35] Hughes AEO, Magrini V, Demeter R, Miller CA, Fulton R, Fulton LL (2014). Clonal architecture of secondary acute myeloid leukemia defined by single-cell sequencing. PLoS Genet..

[36] Macaulay IC, Haerty W, Kumar P, Li YI, Hu TX, Teng MJ (2015). G&T-seq: parallel sequencing of single-cell genomes and transcriptomes. Nat Methods..

[37] Neves RPL, Raba K, Schmidt O, Honisch E, Meier-Stiegen F, Behrens B (2014). Genomic high-resolution profiling of single CK^pos^/CD45^neg^ flow-sorting purified circulating tumor cells from patients with metastatic breast cancer. Clin Chem..

[38] Paolillo C, Mu Z, Rossi G, Schiewer MJ, Nguyen T, Austin L (2017). Detection of activating estrogen receptor gene (ESR1) mutations in single circulating tumor cells. Clin Cancer Res..

[39] Rohrback S, April C, Kaper F, Rivera RR, Liu CS, Siddoway B (2018). Submegabase copy number variations arise during cerebral cortical neurogenesis as revealed by single-cell whole-genome sequencing. Proc Natl Acad Sci USA..

[40] Vitak SA, Torkenczy KA, Rosenkrantz JL, Fields AJ, Christiansen L, Wong MH (2017). Sequencing thousands of single-cell genomes with combinatorial indexing. Nat Methods..

[41] Wang Y, Waters J, Leung ML, Unruh A, Roh W, Shi X (2014). Clonal evolution in breast cancer revealed by single nucleus genome sequencing. Nature..

[42] Zahn H, Steif A, Laks E, Eirew P, VanInsberghe M, Shah SP (2017). Scalable whole-genome single-cell library preparation without preamplification. Nature Methods..

[43] Burbulis IE, Wierman MB, Wolpert M, Haakenson M, Lopes M-B, Schiff D (2018). Improved molecular karyotyping in glioblastoma. Mutat Res.

[44] Campbell IM, Shaw CA, Stankiewicz P, Lupski JR (2015). Somatic mosaicism: implications for disease and transmission genetics. Trends Genet..

[45] Zong C, Lu S, Chapman AR, Xie XS (2012). Genome-wide detection of single-nucleotide and copy-number variations of a single human cell. Science..

[46] Ning L, Li Z, Wang G, Hu W, Hou Q, Tong Y (2015). Quantitative assessment of single-cell whole genome amplification methods for detecting copy number variation using hippocampal neurons. Sci Rep.

[47] Navin N, Kendall J, Troge J, Andrews P, Rodgers L, McIndoo J (2011). Tumour evolution inferred by single-cell sequencing. Nature..

[48] McConnell MJ, Lindberg MR, Brennand KJ, Piper JC, Voet T, Cowing-Zitron C (2013). Mosaic copy number variation in human neurons. Science..

[49] Fu Y, Li C, Lu S, Zhou W, Tang F, Xie XS (2015). Uniform and accurate single-cell sequencing based on emulsion whole-genome amplification. Proc Natl Acad Sci USA..

[50] Gardner MJ, Hall N, Fung E, White O, Berriman M, Hyman RW (2002). Genome sequence of the human malaria parasite *Plasmodium falciparum*. Nature..

[51] Nkhoma SC, Trevino SG, Gorena KM, Nair S, Khoswe S, Jett C (2020). Co-transmission of related malaria parasite lineages shapes within-host parasite diversity. Cell Host Microbe.

[52] Lasken RS, Stockwell TB (2007). Mechanism of chimera formation during the multiple displacement amplification reaction. BMC Biotechnol..

[53] Huckaby AC, Granum CS, Carey MA, Szlachta K, Al-Barghouthi B, Wang Y-H (2018). Complex DNA structures trigger copy number variation across the *Plasmodium falciparum* genome. Nucleic Acids Res.

[54] Simam J, Rono M, Ngoi J, Nyonda M, Mok S, Marsh K (2018). Gene copy number variation in natural populations of *Plasmodium falciparum* in Eastern Africa. BMC Genomics..

[55] Oyola SO, Manske M, Campino S, Claessens A, Hamilton WL, Kekre M (2014). Optimized whole-genome amplification strategy for extremely AT-biased template. DNA Res..

[56] de Bourcy CFA, De Vlaminck I, Kanbar JN, Wang J, Gawad C, Quake SR (2014). A quantitative comparison of single-cell whole genome amplification methods. PLoS One..

[57] Haynes JD, Diggs CL, Hines FA, Desjardins RE (1976). Culture of human malaria parasites *Plasmodium falciparum*. Nature..

[58] Bei AK, Desimone TM, Badiane AS, Ahouidi AD, Dieye T, Ndiaye D (2010). A flow cytometry-based assay for measuring invasion of red blood cells by *Plasmodium falciparum*. Am J Hematol..

[59] Brown AC, Moore CC, Guler JL (2020). Cholesterol-dependent enrichment of understudied erythrocytic stages of human Plasmodium parasites. Sci Rep.

[60] Maniatis T, Sambrook J, Fritsch EF (1989). Molecular cloning: a laboratory manual.

[61] Ribaut C, Berry A, Chevalley S, Reybier K, Morlais I, Parzy D (2008). Concentration and purification by magnetic separation of the erythrocytic stages of all human Plasmodium species. Malar J..

[62] Jensen MA, Fukushima M, Davis RW (2010). DMSO and betaine greatly improve amplification of GC-rich constructs in de novo synthesis. PLOS ONE..

[63] Pickard AL, Wongsrichanalai C, Purfield A, Kamwendo D, Emery K, Zalewski C (2003). Resistance to antimalarials in Southeast Asia and genetic polymorphisms in pfmdr1. Antimicrob Agents Chemother..

[64] Perandin F, Manca N, Calderaro A, Piccolo G, Galati L, Ricci L (2004). Development of a real-time PCR assay for detection of *Plasmodium falciparum*, Plasmodium vivax, and Plasmodium ovale for routine clinical diagnosis. J Clin Microbiol..

[65] Dean FB, Hosono S, Fang L, Wu X, Faruqi AF, Bray-Ward P (2002). Comprehensive human genome amplification using multiple displacement amplification. Proc Natl Acad Sci USA..

[66] Cowman AF, Galatis D, Thompson JK (1994). Selection for mefloquine resistance in *Plasmodium falciparum* is linked to amplification of the pfmdr1 gene and cross-resistance to halofantrine and quinine. Proc Natl Acad Sci U S A..

[67] Bushnell B (2019). BBMap.

[68] Chiang C, Layer RM, Faust GG, Lindberg MR, Rose DB, Garrison EP (2015). SpeedSeq: ultra-fast personal genome analysis and interpretation. Nat Methods..

[69] Li H, Handsaker B, Wysoker A, Fennell T, Ruan J, Homer N (2009). The Sequence Alignment/Map format and SAMtools. Bioinformatics..

[70] Sims D, Sudbery I, Ilott NE, Heger A, Ponting CP (2014). Sequencing depth and coverage: key considerations in genomic analyses. Nat Revi Genet..

[71] García-Alcalde F, Okonechnikov K, Carbonell J, Cruz LM, Götz S, Tarazona S (2012). Qualimap: evaluating next-generation sequencing alignment data. Bioinformatics..

[72] Quinlan AR, Hall IM (2010). BEDTools: a flexible suite of utilities for comparing genomic features. Bioinformatics..

[73] Krzywinski M, Schein J, Birol I, Connors J, Gascoyne R, Horsman D (2009). Circos: an information aesthetic for comparative genomics. Genome Res..

[74] Cheong W-H, Tan Y-C, Yap S-J, Ng K-P (2015). ClicO FS: an interactive web-based service of Circos. Bioinformatics..

[75] Chen C, Xing D, Tan L, Li H, Zhou G, Huang L (2017). Single-cell whole-genome analyses by Linear Amplification via Transposon Insertion (LIANTI). Science..

[76] Marwick B, Krishnamoorthy K. Cvequality: tests for the equality of coefficients of variation from multiple groups. R software package version 0.2.0. https://github.com/benmarwick/cvequality. Accessed 1 Oct 2019.

[77] Chen D, Zhen H, Qiu Y, Liu P, Zeng P, Xia J (2018). Comparison of single cell sequencing data between two whole genome amplification methods on two sequencing platforms. Sci Rep..

[78] Harrell F. E. Hmisc: Harrell miscellaneous. R package Version 4.3-0. https://CRAN.R-project.org/package=Hmisc. Accessed 1 May 2019.

[79] Warnes GR, Bolker B, Bonebakker L, Gentleman R, Liaw WHA, Lumley T, et al. Gplots: Various R programming tools for plotting data. R package version 3.0.1.1. https://cran.r-project.org/web/packages/gplots/index.html. Accessed 1 Oct 2019.

[80] Otto TD, Böhme U, Sanders M, Reid A, Bruske EI, Duffy CW (2018). Long read assemblies of geographically dispersed *Plasmodium falciparum* isolates reveal highly structured subtelomeres. Wellcome Open Res..

[81] Layer RM, Chiang C, Quinlan AR, Hall IM (2014). LUMPY: a probabilistic framework for structural variant discovery. Genome Biol.

[82] Garvin T, Aboukhalil R, Kendall J, Baslan T, Atwal GS, Hicks J (2015). Interactive analysis and assessment of single-cell copy-number variations. Nat Methods..

[83] MalariaGEN, Ahouidi A, Ali M, Almagro-Garcia J, Amambua-Ngwa A, Amaratunga C, et al. An open dataset of Plasmodium falciparum genome variation in 7,000 worldwide samples. Wellcome Open Res. 2021;6:42–42. 10.12688/wellcomeopenres.16168.1 PMC8008441 33824913

[84] MalariaGEN P. falciparum Community Project V6.0 pipeline. ftp://ngs.sanger.ac.uk/production/malaria/pfcommunityproject/Pf6/Pf_6_extended_methods.pdf.

[85] McKenna A, Hanna M, Banks E, Sivachenko A, Cibulskis K, Kernytsky A (2010). The Genome Analysis Toolkit: a MapReduce framework for analyzing next-generation DNA sequencing data. Genome Res..

[86] DePristo MA, Banks E, Poplin R, Garimella KV, Maguire JR, Hartl C (2011). A framework for variation discovery and genotyping using next-generation DNA sequencing data. Nat Genet.

[87] Van der Auwera GA, Carneiro MO, Hartl C, Poplin R, Del Angel G, Levy-Moonshine A (2013). From FastQ data to high confidence variant calls: the Genome Analysis Toolkit best practices pipeline. Curr Protoc Bioinformatics..

[88] Miles A, Iqbal Z, Vauterin P, Pearson R, Campino S, Theron M (2016). Indels, structural variation, and recombination drive genomic diversity in *Plasmodium falciparum*. Genome Res..

[89] Hamilton WL, Claessens A, Otto TD, Kekre M, Fairhurst RM, Rayner JC (2017). Extreme mutation bias and high AT content in *Plasmodium falciparum*. Nucleic Acids Res..

[90] Cingolani P, Platts A, Wang LL, Coon M, Nguyen T, Wang L, et al. A program for annotating and predicting the effects of single nucleotide polymorphisms, SnpEff: SNPs in the genome of Drosophila melanogaster strain w1118; iso-2; iso-3. Fly (Austin). 2012;6:80–92. 10.4161/fly.19695 PMC3679285 22728672

[91] Tollefson GA, Schuster J, Gelin F, Agudelo A, Ragavendran A, Restrepo I (2019). VIVA (VIsualization of VAriants): a VCF file visualization tool. Sci Rep.

[92] Auburn S, Campino S, Clark TG, Djimde AA, Zongo I, Pinches R (2011). An effective method to purify *Plasmodium falciparum* DNA directly from clinical blood samples for whole genome high-throughput sequencing. PLoS One..

[93] Oyola SO, Gu Y, Manske M, Otto TD, O’Brien J, Alcock D (2013). Efficient depletion of host DNA contamination in malaria clinical sequencing. J Clin Microbiol..

[94] Zhang X, Liang B, Xu X, Zhou F, Kong L, Shen J, et al. The comparison of the performance of four whole genome amplification kits on ion proton platform in copy number variation detection. Biosci Rep. 2017;37. 10.1042/BSR20170252. 10.1042/BSR20170252 PMC6434089 28572171

[95] Corneveaux JJ, Kruer MC, Hu-Lince D, Ramsey KE, Zismann VL, Stephan DA (2007). SNP-based chromosomal copy number ascertainment following multiple displacement whole-genome amplification. BioTech..

[96] Arriola E, Lambros MBK, Jones C, Dexter T, Mackay A, Tan DSP (2007). Evaluation of Phi29-based whole-genome amplification for microarray-based comparative genomic hybridisation. Lab Investig..

[97] Chen M, Song P, Zou D, Hu X, Zhao S, Gao S (2014). Comparison of multiple displacement amplification (MDA) and multiple annealing and looping-based amplification cycles (MALBAC) in Single-Cell Sequencing. PLoS One..

[98] Scherf A, Carter R, Petersen C, Alano P, Nelson R, Aikawa M (1992). Gene inactivation of Pf11-1 of *Plasmodium falciparum* by chromosome breakage and healing: identification of a gametocyte-specific protein with a potential role in gametogenesis. EMBO J..

[99] Pirooznia M, Goes FS, Zandi PP (2015). Whole-genome CNV analysis: advances in computational approaches. Front Genet..

[100] Volkman SK, Sabeti PC, DeCaprio D, Neafsey DE, Schaffner SF, Milner DA (2007). A genome-wide map of diversity in *Plasmodium falciparum*. Nat Genet.

[101] Wang Y, Gao Z, Xu Y, Li G, He L, Qian P (2016). An evaluation of multiple annealing and looping based genome amplification using a synthetic bacterial community. The Chin Soc Oceanography..

[102] Ohkubo S, Muto A, Kawauchi Y, Yamao F, Osawa S (1987). The ribosomal protein gene cluster of Mycoplasma capricolum. Mol Gen Genet MGG..

[103] Andersson SGE, Zomorodipour A, Andersson JO, Sicheritz-Pontén T, Alsmark UCM, Podowski RM (1998). The genome sequence of Rickettsia prowazekii and the origin of mitochondria. Nature..

[104] Fraser CM, Casjens S, Huang WM, Sutton GG, Clayton R, Lathigra R (1997). Genomic sequence of a Lyme disease spirochaete, Borrelia burgdorferi. Nature.

[105] Lorenzi HA, Puiu D, Miller JR, Brinkac LM, Amedeo P, Hall N (2010). New assembly, reannotation and analysis of the Entamoeba histolytica genome reveal new genomic features and protein content information. PLoS Negl Trop Dis..

[106] Ohama T, Muto A, Osawa S (1990). Role of GC-biased mutation pressure on synonymous codon choice in Micrococcus luteus a bacterium with a high genomic GC-content. Nucleic Acids Res..

[107] Lasken RS (2013). Single-cell sequencing in its prime. Nat Biotechnol..

[108] Viguera E, Canceill D, Ehrlich SD (2001). In vitro replication slippage by DNA polymerases from thermophilic organisms. J Mol Biol.

[109] Ignatov KB, Barsova EV, Fradkov AF, Blagodatskikh KA, Kramarova TV, Kramarov VM (2014). A strong strand displacement activity of thermostable DNA polymerase markedly improves the results of DNA amplification. Biotechniques..

[110] Srisutham S, Suwannasin K, Mathema VB, Sriprawat K, Smithuis FM, Nosten F (2020). Utility of *Plasmodium falciparum* DNA from rapid diagnostic test kits for molecular analysis and whole genome amplification. Malar J.

[111] Carey MA, Covelli V, Brown A, Medlock GL, Haaren M, Cooper JG (2018). Influential parameters for the analysis of intracellular parasite metabolomics. mSphere.

[112] Waldvogel Abramowski S, Tirefort D, Lau P, Guichebaron A, Taleb S, Modoux C (2018). Cell-free nucleic acids are present in blood products and regulate genes of innate immune response. Transfusion..

[113] Manske M, Miotto O, Campino S, Auburn S, Almagro-Garcia J, Maslen G (2012). Analysis of *Plasmodium falciparum* diversity in natural infections by deep sequencing. Nature..

[114] Jacob CG, Tan JC, Miller BA, Tan A, Takala-Harrison S, Ferdig MT (2014). A microarray platform and novel SNP calling algorithm to evaluate *Plasmodium falciparum* field samples of low DNA quantity. BMC Genomics..

[115] Woyke T, Sczyrba A, Lee J, Rinke C, Tighe D, Clingenpeel S (2011). Decontamination of MDA reagents for single cell whole genome amplification. PLoS One..

[116] Salter SJ, Cox MJ, Turek EM, Calus ST, Cookson WO, Moffatt MF (2014). Reagent and laboratory contamination can critically impact sequence-based microbiome analyses. BMC Biol.

[117] Rand KH, Houck H (1990). Taq polymerase contains bacterial DNA of unknown origin. Mol Cell Probes..

[118] Kil E-J, Kim S, Lee Y-J, Kang E-H, Lee M, Cho S-H (2015). Advanced loop-mediated isothermal amplification method for sensitive and specific detection of Tomato chlorosis virus using a uracil DNA glycosylase to control carry-over contamination. J Virol Methods..

[119] McFeters GA, Broadaway SC, Pyle BH, Egozy Y (1993). Distribution of bacteria within operating laboratory water purification systems. Appl Environ Microbiol..

[120] Kulakov LA, McAlister MB, Ogden KL, Larkin MJ, O’Hanlon JF (2002). Analysis of bacteria contaminating ultrapure water in industrial systems. Appl Environ Microbiol..

[121] Nogami T, Ohto T, Kawaguchi O, Zaitsu Y, Sasaki S (1998). Estimation of bacterial contamination in ultrapure water: application of the anti-DNA antibody. Anal Chem..

[122] Zhang L, Vijg J (2018). Somatic mutagenesis in mammals and its implications for human disease and aging. Annu Rev Genet..

[123] Zhang ZD, Du J, Lam H, Abyzov A, Urban AE, Snyder M (2011). Identification of genomic indels and structural variations using split reads. BMC Genomics..

[124] Hou Y, Fan W, Yan L, Li R, Lian Y, Huang J (2013). Genome analyses of single human oocytes. Cell..

[125] McDaniels JM, Huckaby AC, Carter SA, Lingeman S, Francis A, Congdon M, et al. Extrachromosomal DNA amplicons in antimalarial-resistant *Plasmodium falciparum*. Mol Microbiol. 2020. 10.1111/mmi.14624. 10.1111/mmi.14624 PMC8246734 33053232

[126] Claessens A, Hamilton WL, Kekre M, Otto TD, Faizullabhoy A, Rayner JC (2014). Generation of antigenic diversity in *Plasmodium falciparum* by structured rearrangement of var genes during mitosis. PLoS Genet.

[127] McDew-White M, Li X, Nkhoma SC, Nair S, Cheeseman I, Anderson TJC (2019). Mode and tempo of microsatellite length change in a malaria parasite mutation accumulation experiment. Genome Biol Evol.

[128] Liu S, Huckaby AC, Brown AC, Moore CC, Burbulis I, McConnell MJ, et al. Single cell sequencing of the small and AT-skewed genome of malaria parasites. Github. 2021; https://github.com/Pfal-analysis/Single-cell-sequencing-data. 10.1186/s13073-021-00889-9 PMC8094492 33947449

[129] Liu S, Huckaby AC, Brown AC, Moore CC, Burbulis I, McConnell MJ, Güler JL. Single cell sequencing of the small and AT-skewed genome of malaria parasites. BioProject PRJNA607987, NCBI Sequence Read Archive 2020. https://www.ncbi.nlm.nih.gov/sra/PRJNA607987. 10.1186/s13073-021-00889-9 PMC8094492 33947449

